# Misconceptions and do-not-resuscitate preferences of healthcare professionals commonly involved in cardiopulmonary resuscitations: A national survey

**DOI:** 10.1016/j.resplu.2024.100575

**Published:** 2024-02-13

**Authors:** Simon A. Amacher, Sebastian Gross, Christoph Becker, Armon Arpagaus, Tabita Urben, Jens Gaab, Christian Emsden, Kai Tisljar, Raoul Sutter, Hans Pargger, Stephan Marsch, Sabina Hunziker

**Affiliations:** a Medical Communication and Psychosomatic Medicine, University Hospital Basel, Basel, Switzerland; b Department of Intensive Care, University Hospital Basel, Basel, Switzerland; c Department of Emergency Medicine, University Hospital Basel, Basel, Switzerland; d Division of Clinical Psychology and Psychotherapy, Faculty of Psychology, University of Basel, Switzerland; e Post-Intensive Care Clinic, University Hospital Basel, Basel, Switzerland; f Medical Faculty, University of Basel, Basel, Switzerland

**Keywords:** Cardiac arrest, Cardiopulmonary resuscitation, Ethics, Personal preferences, Shared decision-making, End-of-life care

## Abstract

**Aims:**

To assess the DNR preferences of critical care-, anesthesia- and emergency medicine practitioners, to identify factors influencing decision-making, and to raise awareness for misconceptions concerning CPR outcomes.

**Methods:**

A nationwide multicenter survey was conducted in Switzerland confronting healthcare professionals with a case vignette of an adult patient with an out-of-hospital cardiac arrest (OHCA). The primary outcome was the rate of DNR Code Status vs. CPR Code Status when taking the perspective from a clinical case vignette of a 70-year-old patient. Secondary outcomes were participants’ personal preferences for DNR and estimates of survival with good neurological outcome after in- and out-of-hospital cardiac arrest.

**Results:**

Within 1803 healthcare professionals, DNR code status was preferred in 85% (*n* = 1532) in the personal perspective of the case vignette and 53.2% (*n* = 932) when making a decision for themselves. Main predictors for a DNR Code Status regarding the case vignette included preferences for DNR Code Status for themselves (n [%] 896 [58.5] vs. 87 [32.1]; adjusted odds ratio [OR] 2.97, 95% confidence interval [CI] 2.25–3.92; *p* < 0.001) and lower estimated OHCA survival (mean [±SD] 12.3% [±11.8] vs. 14.7%[±12.8]; adjusted OR 0.98, 95% CI 0.97–0.99; *p* = 0.001). Physicians chose a DNR order more often when compared to nurses and paramedics.

**Conclusions:**

The estimation of outcomes following cardiac arrest and personal living conditions are pivotal factors influencing code status preferences in healthcare professionals. Healthcare professionals should be aware of cardiac arrest prognosis and potential implications of personal preferences when engaging in code status- and end-of-life discussions with patients and their relatives.

## Graphical abstract

**Figure:**
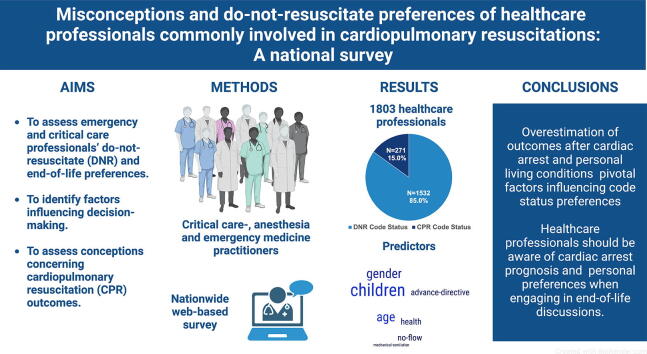

## Introduction

Patients experiencing a cardiac arrest have a high initial mortality, and neurological disability is frequent in survivors.^1^, ^2^, ^3^, ^4^, ^5^ In the 2021 United States of America Heart Disease and Stroke Statistics assessing data from 2019, mortality rates until hospital discharge average 89.5% for out-of-hospital cardiac arrests (OHCA) and 73.3% for in-hospital cardiac arrests (IHCA).^6^ Survivors, however, depict significant rates of neurological impairment leading to partial or complete dependence on others in activities of daily life.^4^, ^5^, ^6^, ^7^, ^8^, ^9^, ^10^ At hospital discharge, around 10–15% of OHCA- and 10–12% of IHCA survivors suffer from severe disability, usually requiring continuous care.^4^, ^5^, ^7^

There is evidence that the public substantially overestimates the success of cardiopulmonary resuscitation (CPR), possibly skewed by the unrealistic display of CPR success in television and movies.^11^, ^12^, ^13^, ^14^, ^15^ The question of whether a patient wishes to be resuscitated or not is commonly discussed on hospital admission in a shared decision-making conversation between the patient and the treating physician, as the hyperacute nature of cardiac arrest makes ad-hoc discussions of resuscitation preferences impossible.^16^ This discussion should be performed upon hospital admission including an assessment of the patient’s health status, information about the individual prognosis in case of a cardiac arrest, potential consequences of a successful resuscitation and take into account patients' advance directives.^16^ Patients' CPR preferences are then usually documented in their medical records, enabling their implementation even when patients are unconscious or otherwise incapable of communicating. This is crucial for patients without readily available next of kin, where physicians often act as surrogate decision-makers.^17^ Thus, health professionals should be aware of their wishes and prejudices concerning CPR to be unbiased counselors for patients in a shared decision-making process or to act as surrogate decision-makers.^18^, ^19^, ^20^ This is especially important in the emergency and critical care setting where end-of-life decisions, and cardiopulmonary resuscitations are prevalent.^21^, ^22^ Hence, it is the aim of this study to assess the DNR preferences of critical care-, anesthesia- and emergency medicine practitioners, to identify factors influencing decision-making, and to assess current conceptions regarding CPR outcomes. The results are then compared to a representative sample of the general population.

## Methods

### Study population

A multicenter web-based survey was conducted in Switzerland among healthcare professionals involved in the care of OHCA and IHCA patients and compared to a representative sample of the general population. In Switzerland, the following healthcare professionals are predominantly involved in advanced cardiac life support and post-resuscitation care:

- Paramedics and prehospital emergency physicians
- Emergency nurses and emergency physicians
- Intensive care nurses and intensive care physicians
- Nurse anesthetists and anesthesiologists

Healthcare professionals or trainees from the aforementioned professions were eligible to be surveyed.

### Survey administration

The national Societies of the respective subspecialties were contacted and asked to participate in the survey. All national societies in question consented to participate in the survey and to distribute the survey link using their email communication channel, except the Swiss Society for Anaesthesiology and Perioperative Medicine, which rejected participation in the survey. To compensate for this matter, anesthesia departments of four large Swiss tertiary care centers (University Hospital Zurich, University Hospital Basel, Cantonal Hospital St. Gallen, and Cantonal Hospital Aarau) were asked to participate instead. Also, as the Swiss paramedics are only incompletely represented in their national society, six large emergency services participated in the survey. A list of all participating societies and institutions can be obtained from the online supplement (Supplement 1). The emails were only sent once without a reminder, and the number of emails sent was registered to calculate the response rate (Supplement 1).

### Questionnaire development

The questionnaire was developed in accordance with the Best Practices for Survey Research^23^ and Strengthening the Reporting of Observational Studies in Epidemiology (STROBE) guidelines.^24^ The questionnaire development has been previously reported in detail^25^; in brief, the multi-stage process involved members of the public (including a hospital pastor) according to the *Patient and Public Involvement* strategy,^26^ senior critical care physicians, a critical care nurse, and a member of the ethical counsel. The final version of the survey can be obtained from the online supplement (Supplement 2).

### Outcomes

The primary outcome was the rate of DNR Code Status vs. CPR Code Status when taking the perspective from a clinical case vignette of a 70-year-old patient suffering an OHCA with a no-flow time (time from collapse to start of CPR)^27^ of 10 minutes (Box 1). The key secondary outcome was the respondents’ personal DNR preferences for themselves, independent of the case vignette.

Box 1 **Clinical Case Vignette for the Primary Outcome.**

Imagine being 70 years old. You have high blood pressure and diabetes. During a walk, you suddenly suffer a cardiac arrest. You lose consciousness and fall to the ground. You don’t breathe anymore, and your heart has also stopped beating. A passerby notices your distress and immediately calls an ambulance, but the person is overwhelmed by the situation and doesn’t take any other measures. After ten minutes, the emergency medical service arrives. Would you want to be resuscitated in this specific situation?

### Baseline characteristics and factors associated with preference for or against cardiopulmonary resuscitation measures

The questionnaire included the following baseline characteristics: age, self-reported gender, language, profession, nationality, specialty and subspecialty, region, highest educational degree, religion, grade of religiousness, comorbidities, years of work experience since degree, living conditions, number of children, and type of emergency service.

Additionally, the following factors were registered:

- Estimated survival rates with independence in activities of daily living after an OHCA or IHCA according to a cerebral performance category scale (CPC) of 1 or 2.^28^, ^29^ In accordance with the original publication, a CPC of 1 indicates “Good recovery - (…) Resumption of normal life even though there may be minor neurological and psychological deficits.” And CPC 2 indicates “Moderate disability - Disabled but independent” in activities of daily living, such as the use of public transportation or doing groceries”.^28^ The survival with independence in activities of daily living estimates were then compared with IHCA and OHCA outcome data from the Heart Disease and Stroke Statistics-2021 Update.^6^ In the Heart Disease and Stroke Statistics-2021 Update, the survival rate until hospital discharge with independence in activities of daily life was reported as 18.0% for IHCA and 8.5% for OHCA.^6^ The responses were categorized as ‘correctly estimated’, ‘underestimated’, and ‘overestimated’ based on a 5% tolerance cut-off in comparison with the data.
- Number of resuscitations performed/participated.
- Refusal of invasive mechanical ventilation in case of severe illness and respiratory failure.
- Possession of an advance directive.
- Maximum time interval after cardiac arrest (no-flow time) after which resuscitation should not be attempted anymore.
- Individual beliefs about an afterlife.
- Previous admission to intensive care.
- Previous admission of a relative to intensive care.
- History of cardiac arrest.
- Perceived self-rating of health measured by the validated EQ-VAS visual analog scale from 0-100.^30^
- Symptoms of anxiety measured by the validated German version of the Generalized Anxiety Disorder-2 questionnaire (GAD-2).^31^
- Symptoms of depression measured by the validated German version of the Patient Health Questionnaire-2 (PHQ-2).^32^

### Statistical analysis

Baseline characteristics and outcomes of healthcare professionals were stratified according to the primary and secondary endpoint. We also compared the health care professionals’ results with the results of a representative of the Swiss general population recently published by our group.^25^ For comparison, we used a two-tailed Student’s t-test.^25^ Logistic and linear regression analyses were used to evaluate associations of these factors with endpoints. Multivariable models were adjusted for age and self-reported gender. Finally, to compare the different professions (i.e., physicians, nurses and paramedics), we used an analysis of variance (ANOVA). A p-value of <0.05 (two-tailed) was considered statistically significant. The statistics software STATA 15.0 (Stata Corp., College Station, TX, USA) was used for all analyses.

## Results

### Baseline characteristics

Of 1822 healthcare professionals participating in the web-based survey (26.5% response rate, Supplement 1) 1803 were included in the final analysis. Of the 1722 healthcare professionals providing information regarding their profession, 812 (47.2%) identified as paramedics, 580 (33.7%) as physicians, and 330 (19.2%) as nurses. In the physician subgroup, 124 participants (21.5%) identified as residents, 180 (34%) as attendings, 168 (31.8%) as consultants, and 57 (10.8%) as heads of department. Average professional experience was (mean [±SD]) 14.2 years [± 10.4]) and 67.7% of participants reported a CPR experience of 21–50 cases Supplement 3.

**Table 1:** Predictors for Code Status preference regarding the case vignette within healthcare professionals.

		All	CPR	DNR	p-value	Unadjusted OR (95%CI)	p-value	Adjusted OR* (95%CI)	p-value
n		1803	271	1532					
*Baseline Characteristics*									
Gender, n (%)	Male	979 (54.3%)	172 (63.5%)	807 (52.7%)	0.001	0.63 (0.48, 0.83)	0.001	0.65 (0.49, 0.85)	0.001

Age, mean (SD)		41.4 (10.6)	42 (10.8)	41.3 (10.6)	0.39	0.99 (0.98, 1.01)	0.387	1 (0.99, 1.01)	0.689

Age categories, n (%)	≤40 years	943 (52.3%)	130 (48.0%)	813 (53.1%)	0.46	1 (ref.)		1 (ref.)	
	41–60 years	788 (43.7%)	128 (47.2%)	660 (43.1%)		0.82 (0.63, 1.07)	0.153	0.63 (0.38, 1.04)	0.072
	61–70 years	62 (3.4%)	11 (4.1%)	51 (3.3%)		0.74 (0.38, 1.46)	0.387	0.48 (0.17, 1.36)	0.165
	>70 years	10 (0.6%)	2 (0.7%)	8 (0.5%)		0.64 (0.13, 3.05)	0.575	0.34 (0.05, 2.33)	0.273

Language, n (%)	German	1519 (84.2%)	198 (73.1%)	1321 (86.2%)	<0.001	1 (ref.)		1 (ref.)	
	French	215 (11.9%)	53 (19.6%)	162 (10.6%)		0.46 (0.32, 0.65)	<0.001	0.48 (0.34, 0.67)	<0.001
	Italian	69 (3.8%)	20 (7.4%)	49 (3.2%)		0.37 (0.21, 0.63)	<0.001	0.39 (0.23, 0.68)	0.001

Religion, n (%)	Don’t know / no information	79 (4.4%)	15 (5.5%)	64 (4.2%)	0.012	1 (ref.)		1 (ref.)	
	No religion	687 (38.1%)	84 (31.0%)	603 (39.4%)		1.68 (0.91, 3.08)	0.095	1.58 (0.86, 2.91)	0.139
	Reformed (Evangelical)	540 (30.0%)	83 (30.6%)	457 (29.8%)		1.41 (0.76, 2.62)	0.282	1.17 (0.63, 2.16)	0.613
	Catholic	478 (26.5%)	82 (30.3%)	396 (25.8%)		1.13 (0.61, 2.08)	0.691	1.05 (0.57, 1.94)	0.873
	Muslim	15 (0.8%)	5 (1.8%)	10 (0.7%)		0.47 (0.14, 1.57)	0.22	0.41 (0.12, 1.4)	0.156
	Other	4 (0.2%)	2 (0.7%)	2 (0.1%)		0.23 (0.03, 1.8)	0.163	0.24 (0.03, 1.83)	0.167

Religiousness, n (%)	Yes	509 (28.2%)	83 (30.6%)	426 (27.8%)	0.34	0.85 (0.63, 1.13)	0.263	0.86 (0.65, 1.15)	0.318

Health self-rating VAS [0–100], mean (SD)	87.5 (11.5)	85.5 (13.2)	87.8 (11.1)	0.003	1.01 (1, 1.02)	0.005	1.01 (1, 1.02)	0.006	

*Experience with cardiac arrest*									
Do you have an advance directive?, n (%)	Yes	563 (32.1%)	56 (21.4%)	507 (34.0%)	<0.001	1.9 (1.39, 2.6)	<0.001	1.95 (1.42, 2.67)	<0.001

Estimated survival**									
Estimated IHCA survival [0–100%], mean (SD)		27.7 (20.3)	27.2 (18.3)	27.7 (20.7)	0.69	1 (0.99, 1.01)	0.673	1 (0.99, 1.01)	0.94

Estimated OHCA survival [0–100%], mean (SD)		12.6 (12)	14.7 (12.8)	12.3 (11.8)	0.002	0.99 (0.98, 1)	0.003	0.98 (0.97, 0.99)	0.001

Estimated IHCA survival (categories), n (%)	Correctly estimated (5% tolerance)	455 (25.2%)	70 (25.8%)	385 (25.1%)	0.78	1 (ref.)		1 (ref.)	
	Underestimated	491 (27.2%)	69 (25.5%)	422 (27.5%)		1.11 (0.78, 1.59)	0.563	1.13 (0.78, 1.61)	0.522
	Overestimated	857 (47.5%)	132 (48.7%)	725 (47.3%)		1.02 (0.74, 1.41)	0.888	0.96 (0.7, 1.32)	0.811

Estimated OHCA survival (categories), n (%)	Correctly estimated (5% tolerance)	1038 (57.6%)	154 (56.8%)	884 (57.7%)	<0.001	1 (ref.)		1 (ref.)	
	Underestimated	225 (12.5%)	15 (5.5%)	210 (13.7%)		2.46 (1.42, 4.27)	0.001	2.39 (1.38, 4.15)	0.002
	Overestimated	540 (30.0%)	102 (37.6%)	438 (28.6%)		0.78 (0.59, 1.03)	0.083	0.7 (0.53, 0.93)	0.013

*Resuscitation preferences*									
Personal resuscitation preference, n (%)	DNR yes	983 (54.5%)	87 (32.1%)	896 (58.5%)	<0.001	2.98 (2.26, 3.95)	<0.001	2.97 (2.25, 3.92)	<0.001

In case of a cardiac arrest: At what time-point without any treatment should resuscitation not be attempted anymore? (categories), n (%)	0–5 min	514 (28.5%)	4 (1.5%)	510 (33.3%)	<0.001	1 (ref.)		1 (ref.)	
	>5–10 min	693 (38.4%)	26 (9.6%)	667 (43.5%)		0.14 (0.05, 0.42)	<0.001	0.21 (0.07, 0.6)	0.004
	>10–15 min	303 (16.8%)	57 (21.0%)	246 (16.1%)		0.03 (0.01, 0.07)	<0.001	0.03 (0.01, 0.09)	<0.001
	>15–60 min	293 (16.3%)	184 (67.9%)	109 (7.1%)		0.01 (0, 0.04)	<0.001	0 (0, 0.01)	<0.001

In the event of severe illness and respiratory failure, would you wish to be mechanically ventilated?, n (%)	NO	1128 (62.6%)	122 (45.0%)	1006 (65.7%)	<0.001	1.96 (1.49, 2.56)	<0.001	2.31 (1.77, 3)	<0.001

*Profession-related information*									
Profession, n (%)	Physician	580 (33.7%)	104 (40.3%)	476 (32.5%)	0.020	1 (ref.)		1 (ref.)	
	Nurse	330 (19.2%)	37 (14.3%)	293 (20.0%)		1.73 (1.16, 2.59)	0.008	1.35 (0.93, 1.96)	0.12
	Paramedic	812 (47.2%)	117 (45.3%)	695 (47.5%)		1.3 (0.97, 1.73)	0.077	1.33 (0.98, 1.79)	0.063

*adjusted for age and self-reported gender.

**With independence in activities of daily living (CPC 1–2).

Abbreviations: CPC, cerebral performance category scale; CPR, cardiopulmonary resuscitation; DNR, do-not-resuscitate; IHCA, in-hospital cardiac arrest; OHCA, out-of-hospital cardiac arrest; OR, odds ratio; ref., reference value; SD, standard deviation VAS, visual analogue scale.

### Primary endpoint: Code Status preference in the personal perspective of the case vignette within healthcare professionals

Regarding the personal perspective of the case vignette, 85% (*n* = 1532) of the 1803 subjects preferred DNR Code Status. The key predictor for a DNR Code Status regarding the case vignette was the OHCA survival estimate: Lower OHCA survival estimates were negatively associated with a DNR Code Status in the case vignette (mean [±SD] 12.3 [±11.8] vs. 14.7 [±12.8]; adjusted odds ratio [OR] 0.98, 95% confidence interval [CI] 0.97–0.99; *p* = 0.001).

Further predictors for a DNR Code Status in the case vignette included DNR Code Status in personal CPR preference, possession of an advance directive, a shorter no-flow time after which resuscitation should not be attempted anymore, not wanting to be mechanically ventilated in case of severe illness and respiratory failure, no belief in an afterlife, no symptoms of anxiety, lower perceived quality of life, and having written an own advance directive (Table 1, Supplement 3).

### Secondary endpoint: Personal Code Status preference among healthcare professionals

Regarding their personal resuscitation preference, 53.2% (*n* = 932) of healthcare professionals preferred a DNR Code Status independent of the circumstances. Main predictors for a personal DNR Code Status included lower estimated IHCA survival (mean [±SD] 26.3 [±19.5] vs. 29.0 [±20.9]; adjusted OR 0.99, 95% CI 0.99–1; *p* = 0.001) and OHCA survival (mean [±SD] 11.4 [±10.6] vs 14.0 [±13.1]; adjusted OR 0.98, 95% CI 0.97–0.99; *p* < 0.001).

Further predictors for a personal DNR Code Status preference included overestimation of OHCA/IHCA survival rates, a shorter no-flow time after which resuscitation should not be attempted anymore, not wanting to be mechanically ventilated in case of severe illness and respiratory failure, not believing in an afterlife, not having children, having an advance directive, and having more professional experience (Supplement 4).

### Interprofessional differences

Regarding the primary outcome, the rate of DNR orders among physicians, nurses, and paramedics was comparable. However, regarding the secondary outcome (personal code status preference), physicians more often chose a DNR order than nurses and paramedics (adjusted OR 0.51, 95% CI 0.39–0.67; *p* < 0.001 and adjusted OR 0.6, 95% CI 0.48–0.75; *p* < 0.001, for nurses and paramedics respectively, Supplement 5). Physicians and paramedics had the highest proportion of correct estimations regarding OHCA outcomes (Fig. 3a, Table 2). When looking at IHCA outcomes, physicians expressed the highest proportion of correct answers (Fig. 2a, Table 2). Also, physicians were less likely to refuse mechanical ventilation than nurses and paramedics (Table 2).

**Table 2:** Interprofessional differences.

		All	Physicians	Nurses	Paramedics	*p-value*
n		1722	580	330	812	
Estimated survival*						
Estimated IHCA survival [0–100%], mean (SD)		27.6 (20.5)	25.7 (17.3)	39.4 (24.5)	24.2 (19.1)	<0.001

Estimated OHCA survival [0–100%], mean (SD)		12.6 (12.1)	10.7 (9.2)	19.3 (16.3)	11.3 (11.1)	<0.001

Estimated IHCA survival (categories), n (%)	Correctly estimated (5% tolerance)	448 (26.0%)	184 (31.7%)	48 (14.5%)	216 (26.6%)	<0.001
	Underestimated	481 (27.9%)	137 (23.6%)	57 (17.3%)	287 (35.3%)	
	Overestimated	793 (46.1%)	259 (44.7%)	225 (68.2%)	309 (38.1%)	

Estimated OHCA survival (categories), n (%)	Correctly estimated (5% tolerance)	985 (57.2%)	358 (61.7%)	126 (38.2%)	501 (61.7%)	<0.001
	Underestimated	221 (12.8%)	81 (14.0%)	28 (8.5%)	112 (13.8%)	
	Overestimated	516 (30.0%)	141 (24.3%)	176 (53.3%)	199 (24.5%)	

Personal resuscitation preference, n (%)	DNR yes	941 (54.6%)	370 (63.8%)	170 (51.5%)	401 (49.4%)	<0.001

In the event of severe illness and respiratory failure, would you wish to be mechanically ventilated?, n (%)	No	1066 (61.9%)	271 (46.7%)	213 (64.5%)	582 (71.7%)	<0.001

*With independence in activities of daily living (CPC 1–2).

Abbreviations: CPC, cereberal performance category scale; DNR, do-not-resuscitate; IHCA, in-hospital cardiac arrest; OHCA, out-of-hospital cardiac arrest; SD, standard deviation.

### Differences between the Swiss general population and healthcare professionals

The Swiss general population cohort included 1044 subjects. The mean age of healthcare professionals was slightly lower than the mean age of the Swiss general population with no difference in gender distribution between the two populations.

**Fig. 1:**
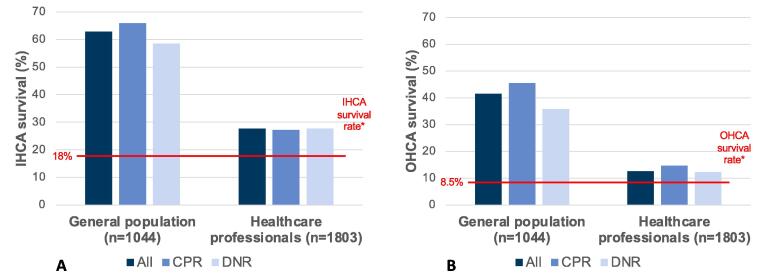
Cardiac arrest survival estimates compared to the actual rate. A. In-hospital cardiac arrest (IHCA) survival estimates compared to the actual rate. *Survival with independence in activities of daily living (CPC 1 or 2) according to Virani et al. (2021)^6^ Abbreviations: CPC, Cerebral Performance Category Scale; IHCA, In-hospital cardiac arrest. B. Out-of-hospital cardiac arrest (OHCA) survival estimates compared to the actual rate. *Survival with independence in activities of daily living (CPC 1 or 2), according to Virani et al. (2021)^6^ Abbreviations: CPC, Cerebral Performance Category Scale; OHCA, Out-of-hospital cardiac arrest.

**Fig. 2:**
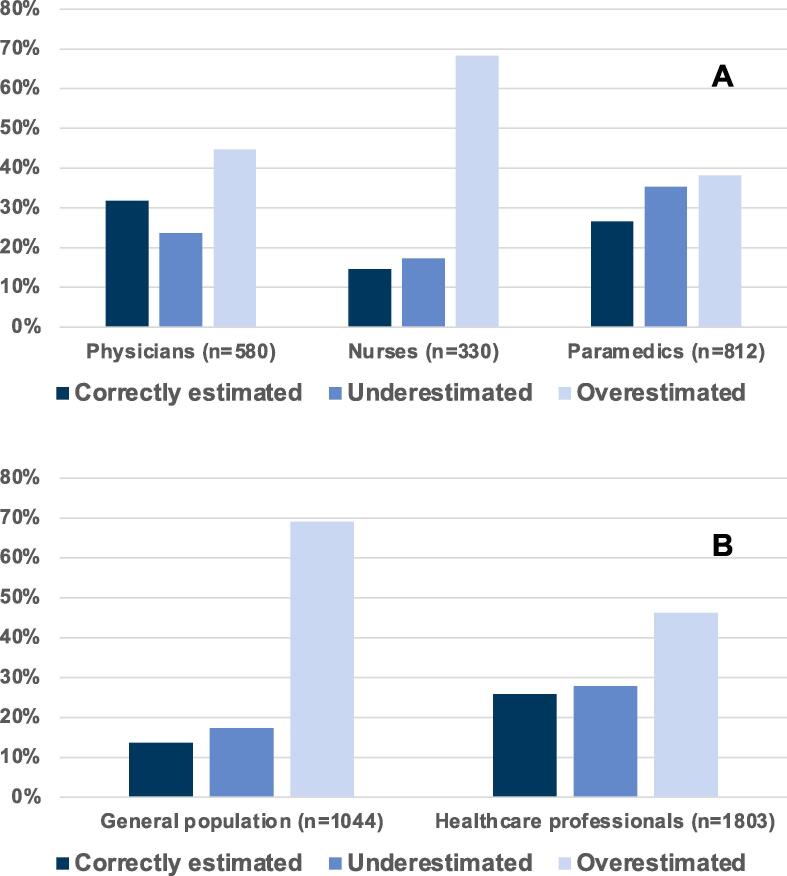
Percentage of correctly estimated, overestimated, and underestimated in-hospital cardiac arrest survival rates. A. Percentage of correctly estimated, overestimated, and underestimated in-hospital cardiac arrest survival rates with independence in activities of daily living (CPC 1–2) among healthcare professionals. Abbreviation: *CPC* Cerebral Performance Category Scale. B. Percentage of correctly estimated, overestimated, and underestimated in-hospital cardiac arrest survival rates with independence in activities of daily living (CPC 1–2) given by the general population and healthcare professionals. Abbreviation: *CPC* Cerebral Performance Category Scale.

**Fig. 3:**
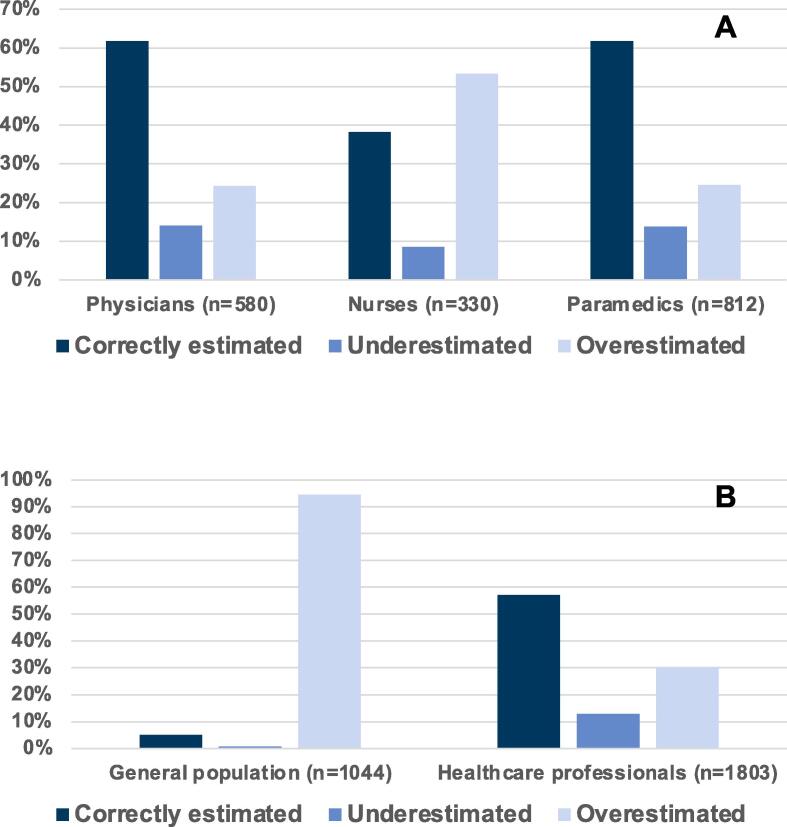
Percentage of correctly estimated, overestimated, and underestimated out-of-hospital cardiac arrest survival rates. A. Percentage of correctly estimated, overestimated, and underestimated out-of-hospital cardiac arrest survival rates with independence in activities of daily living (CPC 1–2) among healthcare professionals. Abbreviation: *CPC* Cerebral Performance Category Scale. B. Percentage of correctly estimated, overestimated, and underestimated out-of-hospital cardiac arrest survival rates with independence in activities of daily living (CPC 1–2) among members of the general population and healthcare professionals. Abbreviation: *CPC* Cerebral Performance Category Scale.

Compared to the Swiss general population, healthcare professionals reported lower IHCA- (mean [±SD] 41.6 [±25.5] vs. 27.7 [±20.6], *p* < 0.001) and OHCA survival estimates (mean [±SD] 62.9 [±25.1] vs. 12.7 [±12.1], *p* < 0.001) (Fig. 1a and 1b). The majority of the general Swiss population overestimated IHCA and OHCA survival probabilities. 57.6% (*n* = 1038) of healthcare professionals estimated OHCA survival correctly (±5%), whereas 12.5% (*n* = 225) underestimated and 30% (*n* = 540) overestimated it. IHCA survival was correctly (±5%) estimated by 25.2% (*n* = 455), underestimated by 27.2% (*n* = 491), and overestimated by 47.5% of subjects (Table 3, Fig. 2b and 3b, Supplement 6). Compared to the Swiss general population, healthcare professionals were less religious, reported fewer symptoms of anxiety and depression, reported a higher mean quality of life, and more often had advance directives (Table 3).

**Table 3:** Differences between the Swiss general population and healthcare professionals.

		All	General Population	Healthcare Professionals	*p-value*
n		2847	1044	1803	
*Baseline Characteristics*					
Gender, n (%)	Male	1503 (52.9%)	528 (50.6%)	975 (54.3%)	0.056

Age, mean (SD)		42.9 (13.1)	45.4 (16.3)	41.437603 (10.609759)	<0.001
Age categories, n (%)	≤40 years	1375 (48.3%)	432 (41.4%)	943 (52.3%)	<0.001
	41–60 years	1202 (42.2%)	414 (39.7%)	788 (43.7%)	
	61–70 years	183 (6.4%)	121 (11.6%)	62 (3.4%)	
	>70 years	87 (3.1%)	77 (7.4%)	10 (0.6%)	

Religiousness, n (%)	Yes	933 (35.0%)	437 (42.9%)	496 (30.1%)	<0.001
Anxiety (GAD-2), n (%)	Yes	229 (8.3%)	121 (11.6%)	108 (6.3%)	<0.001
Depression (PHQ-2), n (%)	Yes	151 (5.5%)	93 (8.9%)	58 (3.4%)	<0.001
Health self-rating VAS [0–100], mean (SD)	84.2 (14.1)	78.9 (16)	87.5 (11.8)	<0.001	
Do you have an advance directive?, n (%)	Yes	846 (30.6%)	283 (28.0%)	563 (32.1%)	0.025

Estimated survival*					
Estimated IHCA survival (categories), n (%)	Correctly estimated (5% tolerance)	594 (21.4%)	139 (13.6%)	455 (25.9%)	<0.001
	Underestimated	668 (24.0%)	177 (17.3%)	491 (27.9%)	
	Overestimated	1517 (54.6%)	706 (69.1%)	811 (46.2%)	

Estimated OHCA survival (categories), n (%)	Correctly estimated (5% tolerance)	1055 (38.1%)	51 (5.0%)	1004 (57.1%)	<0.001
	Underestimated	231 (8.3%)	6 (0.6)	225 (12.8%)	
	Overestimated	1485 (53.6%)	957 (94.4%)	528 (30.1%)	

*Resuscitation preferences*					
Resuscitation preference in the case vignette, n (%)	DNR yes	1955 (68.7%)	423 (40.5%)	1532 (85.0%)	<0.001
Personal resuscitation preference, n (%)	DNR yes	1140 (41.0%)	209 (20.3%)	931 (53.2%)	<0.001
In case of a cardiac arrest: At what time-point without any treatment should resuscitation not be attempted anymore? (min), mean (SD)	12.6 (10.6)	18.3 (13.8)	10 (7.4)	<0.001	

*With independence in activities of daily living (CPC 1–2).

Abbreviations: CPC, cerebral performance category scale; CPR, cardiopulmonary resuscitation; DNR, do-not-resuscitate; GAD-2, Generalized Anxiety Disorder 2-item questionnaire; PHQ-2, Patient Health Questionnaire-2; IHCA, in-hospital cardiac arrest; OHCA, out-of-hospital cardiac arrest; SD, standard deviation.

## Discussion

In this multicenter survey of 1803 healthcare professionals commonly involved in cardiopulmonary resuscitations, a preference for DNR Code Status was found in 85% when taking the perspective of a 70-year-old patient with a substantial no-flow time of 10 minutes. Among the different professions (physicians, nurses, and paramedics) the rate of DNR orders in the personal perspective of the case vignette was comparable. When making a general personal decision, more than half of the healthcare professionals preferred a DNR Code Status for themselves. Notably, physicians more often chose a DNR order for themselves when compared to nurses and paramedics. The proportion of DNR Code Status was significantly higher among healthcare professionals when compared to the general population. This was true for both the case vignette and when making a personal decision for themselves. One could hypothesize that this difference might result from the frequent direct confrontation of healthcare professionals with poor outcomes after cardiac arrest. This is in line with comparable research in the field. Previous findings suggest that there might be substantial discrepancies between what healthcare professionals assume to be a reasonable treatment for themselves and what is considered reasonable for their patients when making clinical decisions involving invasive and burdensome treatments.^33^, ^34^, ^35^ For example, in a recent Australian survey including 747 doctors and 233 nurses approximately 25% of ICU practitioners indicated continuing aggressive treatment for a hypothetical patient. Still, they would refuse the same treatment for themselves.^36^ This might lead to a substantial ethical dilemma between own beliefs, expectations and patients' preferences. In particular when providing perceived futile treatments such ethical dilemmas might cause moral distress for intensive care practitioners, potentially resulting in symptoms of burnout or a change of profession.^21^, ^37^, ^38^ Also, clinicians should keep in mind that many of the functional states (e.g., bowel/bladder incontinence or confinement in bed) commonly observed after critical illness are considered worse than death by a significant number of patients.^39^

In accordance with previously published results from a representative sample of the Swiss general population, healthcare professionals commonly involved in CPR over- and underestimated the survival rate with independence in activities of daily living after cardiac arrest.^25^ Although the surveyed healthcare professionals had a high exposure towards CPR, they substantially overestimated the no-flow time, after which resuscitation should not be attempted anymore. Approximately one third of healthcare professionals set the no-flow time cut-off for not attempting CPR anymore at >10 minutes, although, it is known that a no-flow time of around 10 minutes is associated with a <2% chance of survival without neurological sequelae, depending on the low-flow time.^27^ Still, compared to the general population, healthcare professionals gave lower cardiac arrest survival estimates and more often estimated survival chances correctly.

Interestingly, whilst over- and underestimation of survival rates and refusal of mechanical ventilation were predictive for a DNR Code Status the majority of healthcare professionals did not want to receive invasive mechanical ventilation in case of severe illness and respiratory failure. Healthcare professionals should be well aware of these issues when counseling patients regarding DNR preferences and end-of-life decisions, as poor prognostic estimation, lack of communication skills, and physicians’ attitudes toward death have been shown to interfere with modern end-of-life care.^40^

Interestingly, although intensivists and critical care societies advocate completing an advance directive, only 32.4% of healthcare professionals in our survey possessed an own advance directive.^41^ Notably, possessing an advance directive was predictive for a DNR Code Status, which might indicate previous personal engagement with this topic.

The present study has several implications for clinical practice, personal reflections, and future research. First, healthcare professionals should be aware of their prejudices, choices, and ethical values when supporting patients and families in end-of-life discussions, such as code status preferences. This might potentially influence their counseling and shared decision-making. Standardized communication tools might be supportive in such situations. Currently, a multicenter trial assessing a checklist-guided shared decision-making process performed by our research group has just completed recruitment (https://classic.clinicaltrials.gov/ct2/show/NCT03872154).

Second, healthcare professionals should be aware that a reasonable number of professionals wrongly estimated survival with independence in activities of daily living and overestimated the duration of a reasonable no-flow interval. Thus, we advocate that healthcare professionals commonly counseling patients regarding code status and deciding about termination of CPR are aware of realistic outcome data and time intervals. Third, we suggest that healthcare professionals commonly involved in cardiopulmonary resuscitations engage personally and in-depth with advance directives, as only a minority of the surveyed healthcare professionals possess an advance directive. Additionally, as shown in the present study, previous mental engagement with the topic might influence personal decision-making.

### Strengths and limitations

The present study has several strengths: First, to the best of our knowledge, it is the largest of its kind looking at healthcare professionals’ DNR preferences and comparing them to the preferences of a representative sample of the general population. Second, the present survey was developed in a multi-level iterative process applying the concepts of public and patient involvement and multi-expert input. Also, validated tools for the assessment of anxiety and quality of life were used.^31^, ^32^ Third, the study integrates healthcare professionals from different professions and multiple centers and societies, thus resulting in a high external validity.

However, this study also has limitations: First, as the study was performed exclusively in Switzerland, the results might not be extrapolated to different countries or to populations with other cultural backgrounds. Second, the present study’s design is observational, and the results are thus rather hypothesis generating. Third, the response rate of 26.5% might limit the generalizability of our results due to a selection bias.

## Conclusions

Swiss healthcare professionals have a significantly higher preference for a DNR Code Status compared to the general Swiss population in both a hypothetical clinical case vignette and when making a personal decision for themselves. The estimation of outcomes following cardiac arrest and personal living conditions are pivotal factors influencing code status preferences in healthcare professionals. Healthcare professionals should be aware of cardiac arrest prognosis and potential implications of personal preferences when engaging in code status- and end-of-life discussions with patients and their relatives.

## Ethics approval and consent to participate

For formal clarification of responsibility, the competent ethics committee (Ethics Committee of Northern and Central Switzerland) was consulted, which denied the necessity for ethical approval (Req-2021-01439). A short introduction, including an explanation of the study’s goals and a confidentiality statement, was included on the first page of the online questionnaire. Also, upon checking a box on the first page of the survey, the participants gave informed consent for participation in the study.

## Consent for publication

Not applicable.

## Availability of data and materials

The datasets generated and/or analysed during the current study are available from the corresponding author upon reasonable request.

## CRediT authorship contribution statement

**Simon A. Amacher:** Writing – review & editing, Writing – original draft, Visualization, Validation, Project administration, Methodology, Investigation, Formal analysis, Data curation, Conceptualization. **Sebastian Gross:** Writing – review & editing, Writing – original draft, Visualization, Methodology, Investigation, Formal analysis, Data curation, Conceptualization. **Christoph Becker:** Writing – review & editing, Methodology, Conceptualization. **Armon Arpagaus:** Writing – review & editing, Methodology, Conceptualization. **Tabita Urben:** Writing – review & editing, Methodology, Conceptualization. **Jens Gaab:** Writing – review & editing, Methodology, Conceptualization. **Christian Emsden:** Writing – review & editing, Methodology, Conceptualization. **Kai Tisljar:** Writing – review & editing, Methodology, Conceptualization. **Raoul Sutter:** Writing – review & editing, Methodology, Conceptualization. **Hans Pargger:** Writing – review & editing, Methodology, Conceptualization. **Stephan Marsch:** Writing – review & editing, Methodology, Conceptualization. **Sabina Hunziker:** Writing – review & editing, Writing – original draft, Methodology, Funding acquisition, Formal analysis, Conceptualization.

## Declaration of competing interest

The authors declare the following financial interests/personal relationships which may be considered as potential competing interests: The authors disclose no potential conflict of interest relevant to this study. Simon Amacher has received grants from the Mach-Gaensslen Foundation Switzerland and the Nora van Meeuwen-Haefliger Foundation of the University of Basel, Switzerland outside the present work. Raoul Sutter has received research grants from the Swiss National Science Foundation (No. 320030_169379), the Research Fund of the University of Basel, the Scientific Society Basel, and the Gottfried Julia Bangerter- Rhyner Foundation. Sabina Hunziker was supported by the Gottfried Julia Bangerter- Rhyner Foundation, the Swiss National Science Foundation (SNSF) and the Swiss Society of General Internal Medicine (SSGIM) during the conduct of the study. Grant References 10001C_192850/1 and 10531C_182422.
