# Supplementary material for: Misconceptions and do-not-resuscitate preferences of healthcare professionals commonly involved in cardiopulmonary resuscitations: A national survey
Source: Resusc Plus. 2024 Feb 13;17:100575. doi: 10.1016/j.resplu.2024.100575 (PMC10875294; doi:10.1016/j.resplu.2024.100575)
Supplement: Supplementary data 1 [file mmc1.pdf]

Supplementary Information

Supplement 1. Participating Societies and Institutions

Name*	Description	Number of survey participants	Response rate
**National Societies**			
Swiss Society of Intensive Care Medicine (SSICM)<br><br>https://www.sgi-ssmi.ch/	National society representing intensive care nurses and physicians.	**250**	**19.5%**
Swiss Society for Emergency and Rescue Medicine (SSERM)<br><br>https://sgnor.ch/	National society representing prehospital and in-hospital emergency physicians.	**332**	**26.2%**
Swiss Paramedic Association (SPA)<br><br>https://www.swissparamedic.ch/	National society representing paramedics.	**515**	**33.1%**
Notfallpflege Schweiz<br><br>https://www.notfallpflege.ch/	National society representing emergency medicine nurses.	**22**	**2.8%**
**Anesthesia Departments**			
Institute of Anesthesiology, University Hospital Zurich, Switzerland	Anesthesiologists, emergency physicians, intensive care physicians and nurse anesthetists	**60**	**30%**
Clinic for Anaesthesia, Intermediate Care, Prehospital Emergency Medicine and Pain Therapy, University Hospital Basel, Switzerland	Anesthesiologists, emergency physicians, intensive care physicians and nurse anesthetists	**83**	**27.5%**

Department of Anesthesia, Intensive Care, Emergency and Pain Medicine,<br>Kantonsspital St. Gallen,<br>Switzerland	Anesthesiologists, emergency physicians, intensive care physicians and nurse anesthetists	**102**	**32.7%**
Department of Anaesthesia,<br>Cantonal Hospital Aarau,<br>Switzerland	Anesthesiologists, emergency physicians, intensive care physicians and nurse anesthetists	**50**	**30.5%**
**Helicopter Rescue and Ambulance Services**			
Swiss Air-Rescue REGA,<br>Kloten, Switzerland	Paramedics and prehospital emergency physicians	**77**	**33.8%**
Ambulance Service Regio 144,<br>Rueti, Switzerland	Paramedics and prehospital emergency physicians	**43**	**46.7%**
Ambulance Service<br>Rettung St. Gallen,<br>St. Gallen, Switzerland	Paramedics and prehospital emergency physicians	**77**	**35.6%**
Ambulance Service<br>Rettung Basel-Stadt,<br>Basel, Switzerland	Paramedics and prehospital emergency physicians	**59**	**45.4%**
Ambulance Service<br>Schutz & Rettung Zürich,<br>Zurich, Switzerland	Paramedics and prehospital emergency physicians	**85**	**42.5%**
Ambulance Service<br>Schutz & Rettung Bern,<br>Berne, Switzerland	Paramedics and prehospital emergency physicians	**48**	**32.2%**

*If no English name is available, the German name is displayed. Information concerning the members of the respective society or institution can be obtained from the description.

Supplement 2. Survey

1. Your age: _____ years
2. Your gender:
    a. Female
    b. Male
    c. Other
    d. No information
3. Imagine being 70 years old. You have high blood pressure and diabetes. During a walk, you suddenly suffer a cardiac arrest. You lose consciousness and fall to the ground. You don't breathe anymore, and your heart has also stopped beating. A passerby notices your distress and immediately calls an ambulance, but the person is overwhelmed by the situation and doesn't take any measures. After 10 minutes, the emergency medical service arrives. Would you want to be resuscitated in this specific situation?
    a. Yes
    b. Probably yes
    c. Probably no
    d. No
    e. Don't know / no information
4. In the event of a cardiac arrest, would you want to be resuscitated regardless of the circumstances?
    a. Yes
    b. Probably yes
    c. Probably no
    d. No
    e. Don't know / no information
5. *If 4. is answered a. or b.:* Imagine you are experiencing cardiac arrest: At what time-period without any treatment should resuscitation not be attempted anymore?

    No resuscitation attempt should be made after ______ [0-60] minutes.

6. In the event of severe illness and respiratory failure, would you wish to be mechanically ventilated?
    a. Yes
    b. Probably yes
    c. Probably no
    d. No
    e. Don't know / no information
7. If you had to decide right now: What option would you chose? *(one answer)*
    a. Prolonging life is more important to me, even if it means more pain and discomfort.
    b. Alleviating pain and discomfort is more important to me, even if this might shorten life
    c. I'm not sure
    d. Don't know / no information
8. In case of imminent death: Where would you like to die? (*one answer*)
    a. At home
    b. At a relative's or friend's house

c. In a hospital
d. In a hospice
e. In a nursing home
f. Doesn't matter
g. Other
h. Don’t know/ no information

9. Please estimate: How many patients survive a cardiopulmonary resuscitation and experience independence in activities of daily living afterwards, if the cardiopulmonary resuscitation takes place inside a hospital?

   Out of 100 patients, ______[0-100] experience independence in activities of daily living .

10. Please estimate: How many patients survive a cardiopulmonary resuscitation and experience independence in activities of daily living afterwards, if the cardiopulmonary resuscitation takes place outside a hospital?

    Out of 100 patients, ______[0-100] experience independence in activities of daily living.

11. Have you completed an advance directive?
    a. Yes
    b. No
    c. Don’t know/ no information
12. What is your primary profession?
    a. Physician
    b. Nurse
    c. Paramedic
    d. Emergency medical technician
    e. Don’t know/ no information
13. *If 12. answered a.:* What is your position?
    a. Resident
    b. Attending
    c. Consultant
    d. Head of Department
    e. Don’t know/ no information
14. *If 12. answered a.:* Have you completed any of the following postgraduate courses? (multiple choice)
    a. Emergency care
    b. Intensive care
    c. Anesthesia care
    d. None
    e. Don’t know/ no information
15. In which fields are you predominantly active? (*multiple choice*)
    a. Intensive care
    b. Anesthesia
    c. Emergency (clinical)
    d. Emergency (preclinical)
    e. Internal medicine
    f. Surgery

        g. Don’t know/ no information

16. *If 12. answered c. or d.:* In what kind of emergency medical service do you work predominantly?
    a. Urban area
    b. Rural Area
    c. Air rescue
    d. Don’t know/ no information
17. Do you work in a managerial position?
    a. Yes
    b. No
    c. Don’t know/ no information
18. Years of professional experience (if you are still in training, please indicate “0”): _____ years
19. How many resuscitations have you been involved in?
    a. None
    b. 1-5
    c. 6-10
    d. 11-20
    e. 21-50
    f. >50
20. What is your living situation? (*multiple choice*)
    a. I live alone
    b. I live together with my partner
    c. I live together with my children
    d. I live together with a parent/ my parents
    e. I live together with a sibling/ my siblings
    f. I live together with other adults from my family
    g. I live together with other adults (not related)
    h. Don’t know/ no information
21. Have you got children?
    a. Yes
    b. No
    c. Don’t know/ no information
22. *If 21. answered yes:* How many children of the following ages have you got?
    a. <10 years:
        i. 1 child
        ii. 2 children
        iii. 3 or more children
        iv. None
    b. 10 to 18 years:
        i. 1 child
        ii. 2 children
        iii. 3 or more children
        iv. None
    c. >18 years:
        i. 1 child
        ii. 2 children
        iii. 3 or more children

iv. None

23. Your nationality:
    a. Albania
    b. Belgium
    c. Bosnia and Herzegovina
    d. Germany
    e. France
    f. Greece
    g. Italy
    h. Kosovo
    i. Croatia
    j. Montenegro
    k. Netherlands
    l. North Macedonia
    m. Austria
    n. Portugal
    o. Switzerland
    p. Serbia
    q. Spain
    r. Turkey
    s. Other (please specify): _______
    t. Don't know/ no information
24. Your mother language:
    a. German
    b. French
    c. Italian
    d. English
    e. Other (please specify): _______
    f. Don't know/ no information
25. What religion are you affiliated with?
    a. No religion
    b. Protestant (Evangelical)
    c. Catholic
    d. Jewish
    e. Muslim
    f. Hindu
    g. Other (please specify): _______
    h. Don't know/ no information
26. *If 25. answered b.-g.:* How religious would you describe yourself to be?
    a. Very religious
    b. Somewhat religious
    c. Not religious
    d. Not religious at all
    e. Don't know/ no information
27. What is your belief regarding an afterlife?
    a. Rebirth (reincarnation)
    b. Only the body dies, the soul lives on.

    c. With the death of a human being body and soul die. Nothing remains.
    d. Don’t know/ no information

28. Have you ever been admitted to intensive care?
    a. Yes
    b. No
    c. Don’t know/ no information
29. Has someone very close to you ever been admitted to intensive care?
    a. Yes
    b. No
    c. Don’t know/ no information
30. Have you ever been present when somebody was resuscitated?
    a. Yes
    b. No
    c. Don’t know/ no information
31. Have you ever been resuscitated?
    a. Yes
    b. No
    c. Don’t know/ no information
32. Do you suffer, or have you suffered from, any of the following diseases? (*multiple choice*)
    a. Cancer (diagnosed within the past 3 years)
    b. Cardiovascular disease (e.g., high blood pressure, heart attack, congestive heart failure)
    c. Diabetes
    d. Kidney disease (e.g., chronic kidney failure)
    e. Lung or respiratory disease (e.g., asthma or smoker's lung)
    f. Chronic infectious disease (e.g., HIV, hepatitis, tuberculosis)
    g. Mental illness
    h. Neurological diseases (e.g., multiple sclerosis, paralysis)
    i. No previous illnesses
    j. Other (please specify): _______
    k. Don’t know/ no information
33. Generalized Anxiety Disorder Assessment (GAD-2): Over the last 2 weeks, how often have you experienced any of the following problems?
    a. Feeling nervous, anxious, or on edge
        i. Not at all
        ii. Several days
        iii. More than half the days
        iv. Nearly every day
    b. Unable to stop or control worrying
        i. Not at all
        ii. Several days
        iii. More than half the days
        iv. Nearly every day
34. Patient Health Questionnaire (PHQ-2): Over the last 2 weeks, how often have you experienced any of the following problems?
    a. Little interest or pleasure in doing things
        i. Not at all

ii. Several days
iii. More than half the days
iv. Nearly every day

b. Feeling down, depressed, or hopeless
   i. Not at all
   ii. Several days
   iii. More than half the days
   iv. Nearly every day

35. Below is a scale similar to a thermometer to help you assess your current state of health - with the best being “100”, and the worst “0”. We would now like you to indicate on this scale how good or bad you think your health is today.

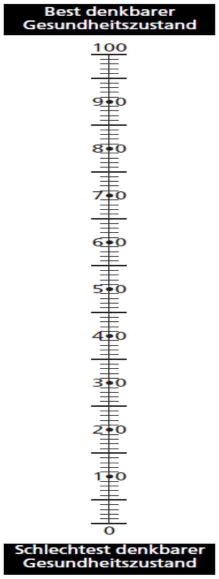

Supplement 3. Predictors for Code Status preference regarding the case vignette within healthcare professionals

		All	CPR	DNR	p-value	Unadjusted OR (95%CI)	p-value	Adjusted OR* (95%CI)	p-value
**n**		1803	271	1532					
Baseline Characteristics									
**Gender, n (%)**	Male	979 (54.3%)	172 (63.5%)	807 (52.7%)	0.001	0.63 (0.48, 0.83)	0.001	0.65 (0.49, 0.85)	0.001
**Age, mean (SD)**		41.4 (10.6)	42 (10.8)	41.3 (10.6)	0.39	0.99 (0.98, 1.01)	0.387	1 (0.99, 1.01)	0.689
**Age categories, n (%)**	≤40 years	943 (52.3%)	130 (48.0%)	813 (53.1%)	0.46	1 (ref.)		1 (ref.)	
	41-60 years	788 (43.7%)	128 (47.2%)	660 (43.1%)		0.82 (0.63, 1.07)	0.153	0.63 (0.38, 1.04)	0.072
	61-70 years	62 (3.4%)	11 (4.1%)	51 (3.3%)		0.74 (0.38, 1.46)	0.387	0.48 (0.17, 1.36)	0.165
	>70 years	10 (0.6%)	2 (0.7%)	8 (0.5%)		0.64 (0.13, 3.05)	0.575	0.34 (0.05, 2.33)	0.273
**Language, n (%)**	German	1519 (84.2%)	198 (73.1%)	1321 (86.2%)	<0.001	1 (ref.)		1 (ref.)	
	French	215 (11.9%)	53 (19.6%)	162 (10.6%)		0.46 (0.32, 0.65)	<0.001	0.48 (0.34, 0.67)	<0.001
	Italian	69 (3.8%)	20 (7.4%)	49 (3.2%)		0.37 (0.21, 0.63)	<0.001	0.39 (0.23, 0.68)	0.001
**Religion, n (%)**	Don't know / no information	79 (4.4%)	15 (5.5%)	64 (4.2%)	0.012	1 (ref.)		1 (ref.)	
	No religion	687 (38.1%)	84 (31.0%)	603 (39.4%)		1.68 (0.91, 3.08)	0.095	1.58 (0.86, 2.91)	0.139
	Reformed (Evangelical)	540 (30.0%)	83 (30.6%)	457 (29.8%)		1.41 (0.76, 2.62)	0.282	1.17 (0.63, 2.16)	0.613
	Catholic	478 (26.5%)	82 (30.3%)	396 (25.8%)		1.13 (0.61, 2.08)	0.691	1.05 (0.57, 1.94)	0.873
	Muslim	15 (0.8%)	5 (1.8%)	10 (0.7%)		0.47 (0.14, 1.57)	0.22	0.41 (0.12, 1.4)	0.156
	Other	4 (0.2%)	2 (0.7%)	2 (0.1%)		0.23 (0.03, 1.8)	0.163	0.24 (0.03, 1.83)	0.167
**Religiousness, n (%)**	Yes	509 (28.2%)	83 (30.6%)	426 (27.8%)	0.34	0.85 (0.63, 1.13)	0.263	0.86 (0.65, 1.15)	0.318
**What is your concept of the afterlife?, n (%)**	Don't know / no information	434 (24.1%)	57 (21.0%)	377 (24.6%)	0.097	1 (ref.)		1 (ref.)	
	Rebirth (reincarnation)	70 (3.9%)	11 (4.1%)	59 (3.9%)		0.77 (0.38, 1.56)	0.466	0.76 (0.38, 1.55)	0.454
	Only the body dies, the soul lives on	672 (37.3%)	119 (43.9%)	553 (36.1%)		0.73 (0.51, 1.03)	0.076	0.64 (0.45, 0.9)	0.011
	When a person dies, body and soul die. There is no survival.	627 (34.8%)	84 (31.0%)	543 (35.4%)		0.98 (0.68, 1.4)	0.901	0.97 (0.67, 1.39)	0.85
**Children, n (%)**	Yes	914 (50.7%)	157 (57.9%)	757 (49.4%)	0.01	0.72 (0.55, 0.94)	0.014	0.75 (0.55, 1)	0.051
**Nationality, n (%)**	Swiss	1496 (83.0%)	215 (79.3%)	1281 (83.6%)	0.065	1 (ref.)		1 (ref.)	
	German	196 (10.9%)	32 (11.8%)	164 (10.7%)		0.81 (0.54, 1.23)	0.32	0.91 (0.61, 1.37)	0.659
	French	15 (0.8%)	6 (2.2%)	9 (0.6%)		0.24 (0.09, 0.69)	0.008	0.26 (0.09, 0.74)	0.011
	Italian	27 (1.5%)	6 (2.2%)	21 (1.4%)		0.57 (0.23, 1.42)	0.227	0.63 (0.25, 1.59)	0.332
	Austrian	25 (1.4%)	3 (1.1%)	22 (1.4%)		1.19 (0.35, 4.01)	0.78	1.16 (0.34, 3.93)	0.81
	Other	44 (2.4%)	9 (3.3%)	35 (2.3%)		0.63 (0.3, 1.33)	0.227	0.66 (0.31, 1.39)	0.271
**Swiss nationality, n (%)**	Yes	1506 (83.5%)	216 (79.7%)	1290 (84.2%)	0.066	1.4 (1.01, 1.95)	0.044	1.31 (0.94, 1.81)	0.109
**Do you suffer or have you suffered from any of the following**	Cancer (diagnosed within the past 3 years)	24 (1.3%)	3 (1.1%)	21 (1.4%)	0.73	1.24 (0.37, 4.19)	0.727	1.26 (0.37, 4.29)	0.708
	Cardiovascular disease (e.g. high blood pressure, heart attack, congestive heart failure)	128 (7.1%)	24 (8.9%)	104 (6.8%)	0.22	0.75 (0.47, 1.19)	0.223	0.84 (0.52, 1.37)	0.491
	Diabetes	16 (0.9%)	3 (1.1%)	13 (0.8%)	0.68	0.76 (0.22, 2.7)	0.677	0.85 (0.24, 3.03)	0.803

**diseases?, n (%)**	Kidney disease (e.g. chronic kidney failure)	9 (0.5%)	1 (0.4%)	8 (0.5%)	0.74	1.42 (0.18, 11.38)	0.743	1.28 (0.16, 10.36)	0.816
	Lung or respiratory disease (e.g. asthma or smoker's lung)	111 (6.2%)	22 (8.1%)	89 (5.8%)	0.14	0.7 (0.43, 1.13)	0.147	0.7 (0.43, 1.13)	0.144
	Chronic infectious disease (e.g. HIV, hepatitis, tuberculosis)	5 (0.3%)	0 (0.0%)	5 (0.3%)	0.35	n.a.	n.a.	n.a.	n.a.
	Mental illness	49 (2.7%)	7 (2.6%)	42 (2.7%)	0.88	1.06 (0.47, 2.39)	0.882	1.02 (0.45, 2.3)	0.963
	Neurological diseases (e.g. multiple sclerosis, paralysis)	14 (0.8%)	1 (0.4%)	13 (0.8%)	0.41	2.31 (0.3, 17.74)	0.421	2.09 (0.27, 16.11)	0.48
	No previous illnesses	1238 (68.7%)	168 (62.0%)	1070 (69.8%)	0.01	1.42 (1.09, 1.86)	0.01	1.38 (1.05, 1.81)	0.021
**Anxiety (GAD-2), n (%)**	Yes	108 (6.0%)	24 (8.9%)	84 (5.5%)	0.031	0.58 (0.36, 0.93)	0.025	0.58 (0.36, 0.93)	0.024
**Depression (PHQ-2), n (%)**	Yes	58 (3.2%)	12 (4.4%)	46 (3.0%)	0.22	0.65 (0.34, 1.25)	0.197	0.69 (0.36, 1.32)	0.258
**Health self-rating VAS [0-100], mean (SD)**		87.5 (11.5)	85.5 (13.2)	87.8 (11.1)	0.003	1.01 (1, 1.02)	0.005	1.01 (1, 1.02)	0.006
Experience with cardiac arrest									
**Have you ever been admitted to an intensive care unit?, n (%)**	Yes	122 (6.8%)	19 (7.0%)	103 (6.7%)	0.86	0.93 (0.56, 1.55)	0.789	1.01 (0.61, 1.69)	0.963
**Has someone very close to you ever been admitted to an intensive care unit?, n (%)**	Yes	1093 (60.6%)	174 (64.2%)	919 (60.0%)	0.19	0.87 (0.66, 1.14)	0.316	0.88 (0.67, 1.16)	0.352
**Have you ever witnessed a cardiopulmonary resuscitation?, n (%)**	Yes	1757 (97.4%)	256 (94.5%)	1501 (98.0%)	<0.001	2.96 (1.57, 5.56)	0.001	3 (1.59, 5.66)	0.001
**Have you ever been resuscitated?, n (%)**	Yes	18 (1.0%)	4 (1.5%)	14 (0.9%)	0.39	0.6 (0.2, 1.84)	0.374	0.68 (0.22, 2.08)	0.495
**Do you have an advance directive?, n (%)**	Yes	563 (32.1%)	56 (21.4%)	507 (34.0%)	<0.001	1.9 (1.39, 2.6)	<0.001	1.95 (1.42, 2.67)	<0.001
Estimated survival**									
**Estimated IHCA survival [0-100%], mean (SD)**		27.7 (20.3)	27.2 (18.3)	27.7 (20.7)	0.69	1 (0.99, 1.01)	0.673	1 (0.99, 1.01)	0.94
**Estimated OHCA survival [0-100%], mean (SD)**		12.6 (12)	14.7 (12.8)	12.3 (11.8)	0.002	0.99 (0.98, 1)	0.003	0.98 (0.97, 0.99)	0.001
**Estimated IHCA survival (categories), n (%)**	Correctly estimated (5% tolerance)	455 (25.2%)	70 (25.8%)	385 (25.1%)	0.78	1 (ref.)		1 (ref.)	
	Underestimated	491 (27.2%)	69 (25.5%)	422 (27.5%)		1.11 (0.78, 1.59)	0.563	1.13 (0.78, 1.61)	0.522

	Overestimated	857 (47.5%)	132 (48.7%)	725 (47.3%)		1.02 (0.74, 1.41)	0.888	0.96 (0.7, 1.32)	0.811
**Estimated OHCA survival (categories), n (%)**	Correctly estimated (5% tolerance)	1038 (57.6%)	154 (56.8%)	884 (57.7%)	<0.001	1 (ref.)		1 (ref.)	
	Underestimated	225 (12.5%)	15 (5.5%)	210 (13.7%)		2.46 (1.42, 4.27)	0.001	2.39 (1.38, 4.15)	0.002
	Overestimated	540 (30.0%)	102 (37.6%)	438 (28.6%)		0.78 (0.59, 1.03)	0.083	0.7 (0.53, 0.93)	0.013
Resuscitation preferences									
**Personal resuscitation preference, n (%)**	DNR yes	983 (54.5%)	87 (32.1%)	896 (58.5%)	<0.001	2.98 (2.26, 3.95)	<0.001	2.97 (2.25, 3.92)	<0.001
**In case of a cardiac arrest: At what time-point without any treatment should resuscitation not be attempted anymore? (min), mean (SD)**		10.4 (6.9)	18.4 (7.2)	9 (5.8)	<0.001	0.87 (0.85, 0.89)	<0.001	0.81 (0.79, 0.83)	<0.001
**In case of a cardiac arrest: At what time-point without any treatment should resuscitation not be attempted anymore? (categories), n (%)**	0-5 min	514 (28.5%)	4 (1.5%)	510 (33.3%)	<0.001	1 (ref.)		1 (ref.)	
	>5-10 min	693 (38.4%)	26 (9.6%)	667 (43.5%)		0.14 (0.05, 0.42)	<0.001	0.21 (0.07, 0.6)	0.004
	>10-15 min	303 (16.8%)	57 (21.0%)	246 (16.1%)		0.03 (0.01, 0.07)	<0.001	0.03 (0.01, 0.09)	<0.001
	>15-60 min	293 (16.3%)	184 (67.9%)	109 (7.1%)		0.01 (0, 0.04)	<0.001	0 (0, 0.01)	<0.001
**In the event of severe illness and respiratory failure, would you wish to be mechanically ventilated?, n (%)**	NO	1128 (62.6%)	122 (45.0%)	1006 (65.7%)	<0.001	1.96 (1.49, 2.56)	<0.001	2.31 (1.77, 3)	<0.001
**If you had to decide now: What option would you prefer?, n (%)**	Don't know / no information	22 (1.2%)	6 (2.2%)	16 (1.0%)	<0.001	1 (ref.)		1 (ref.)	
	Prolonging life is more important to me, even if it means more pain and discomfort	109 (6.0%)	38 (14.0%)	71 (4.6%)		0.7 (0.25, 1.94)	0.493	0.62 (0.22, 1.73)	0.363
	Alleviating pain and discomfort is more important to me, even if this might shorten life	1491 (82.7%)	181 (66.8%)	1310 (85.5%)		2.78 (1.07, 7.19)	0.036	2.41 (0.92, 6.26)	0.072
	I'm not sure what I would choose	181 (10.0%)	46 (17.0%)	135 (8.8%)		1.1 (0.41, 2.98)	0.85	1 (0.37, 2.71)	0.994
**In case of imminent**	Don't know / no information	223 (12.4%)	32 (11.8%)	191 (12.5%)	0.55	1 (ref.)		1 (ref.)	

**death: Where would you like to die?, n (%)**	At home	947 (52.5%)	148 (54.6%)	799 (52.2%)		0.9 (0.6, 1.37)	0.634	0.91 (0.6, 1.38)	0.656
	At a relative's or friend's house	83 (4.6%)	12 (4.4%)	71 (4.6%)		1.9 (0.55, 6.55)	0.31	0.98 (0.48, 2.01)	0.952
	In a hospital	126 (7.0%)	23 (8.5%)	103 (6.7%)		0.75 (0.42, 1.35)	0.337	0.74 (0.41, 1.33)	0.31
	In a hospice	223 (12.4%)	25 (9.2%)	198 (12.9%)		1.33 (0.76, 2.32)	0.322	1.24 (0.7, 2.17)	0.461
	In a nursing home	n.a.	n.a.	n.a.	n.a.	n.a.	n.a.	n.a.	n.a.
	Doesn't matter	201 (11.1%)	31 (11.4%)	170 (11.1%)		0.92 (0.54, 1.57)	0.756	0.95 (0.55, 1.62)	0.845
Profession-related information									
**Profession, n (%)**	Physician	580 (33.7%)	104 (40.3%)	476 (32.5%)	0.020	1 (ref.)		1 (ref.)	
	Nurse	330 (19.2%)	37 (14.3%)	293 (20.0%)		1.73 (1.16, 2.59)	0.008	1.35 (0.93, 1.96)	0.12
	Paramedic	812 (47.2%)	117 (45.3%)	695 (47.5%)		1.3 (0.97, 1.73)	0.077	1.33 (0.98, 1.79)	0.063
**Position, n (%)**	Resident	124 (23.4%)	12 (12.4%)	112 (25.9%)	0.031	1 (ref.)		1 (ref.)	
	Attending	180 (34.0%)	37 (38.1%)	143 (33.1%)		0.41 (0.21, 0.83)	0.013	0.47 (0.23, 1)	0.049
	Consultant	168 (31.8%)	38 (39.2%)	130 (30.1%)		0.37 (0.18, 0.74)	0.005	0.43 (0.18, 1.03)	0.058
	Head of Department	57 (10.8%)	10 (10.3%)	47 (10.9%)		0.5 (0.2, 1.25)	0.138	0.53 (0.17, 1.59)	0.256
**Type of Emergency Medical Service, n (%)**	Urban area	470 (57.3%)	68 (58.1%)	402 (57.2%)	0.73	1 (ref.)		1 (ref.)	
	Rural area	310 (37.8%)	45 (38.5%)	265 (37.7%)		1 (0.66, 1.51)	0.995	1.02 (0.68, 1.54)	0.924
	Air rescue	40 (4.9%)	4 (3.4%)	36 (5.1%)		1.22 (0.46, 3.23)	0.686	1.78 (0.61, 5.23)	0.291
**Managerial position, n(%)**	Yes	665 (36.9%)	112 (41.3%)	553 (36.1%)	0.1	0.89 (0.64, 1.25)	0.503	0.9 (0.67, 1.2)	0.472
**Profession experience (years), mean (SD)**		14.2 (10.4)	15.1 (10.8)	14.1 (10.4)	0.14	0.99 (0.98, 1)	0.137	0.98 (0.95, 1)	0.099
**Number of CPRs**	None	22 (1.2%)	7 (2.6%)	15 (1.0%)	0.093	1 (ref.)		1 (ref.)	
	1 to 5	124 (6.9%)	13 (4.8%)	111 (7.2%)		3.98 (1.37, 11.56)	0.011	3.97 (1.36, 11.58)	0.012
	6 to 10	184 (10.2%)	21 (7.7%)	163 (10.6%)		3.58 (1.31, 9.78)	0.013	3.93 (1.43, 10.83)	0.008
	11 to 20	258 (14.3%)	43 (15.9%)	215 (14.0%)		2.43 (0.93, 6.36)	0.07	2.58 (0.99, 6.77)	0.053
	21 to 50	507 (28.1%)	77 (28.4%)	430 (28.1%)		2.72 (1.07, 6.91)	0.035	3.04 (1.18, 7.82)	0.021
	>50	708 (39.3%)	110 (40.6%)	598 (39.0%)		2.51 (1, 6.31)	0.05	3.34 (1.28, 8.71)	0.013

*adjusted for age and self-reported gender
**With independence in activities of daily living (CPC 1-2).
Abbreviations: CPC , cerebral performance category scale; CPR, cardiopulmonary resuscitation; DNR, do-not-resuscitate; IHCA, in-hospital cardiac arrest; OHCA, out-of-hospital cardiac arrest; OR, odds ratio; ref., reference value; SD, standard deviation VAS, visual analogue scale.

Supplement 4. Predictors for personal Code Status within healthcare professionals

		All	CPR	DNR	*p-value*	Unadjusted OR (95%CI)	*p-value*	Adjusted OR* (95%CI)	*p-value*
**n**		1749	818	931					
Baseline Characteristics									
**Gender, n (%)**	Male	962 (55.0%)	482 (58.9%)	480 (51.6%)	0.002	0.74 (0.61, 0.89)	0.002	0.69 (0.57, 0.83)	<0.001
**Age, mean (SD)**		41.4 (10.6)	40.3 (10.4)	42.5 (10.7)	<0.001	1.02 (1.01, 1.03)	<0.001	1.02 (1.01, 1.03)	<0.001
**Age categories, n (%)**	≤40 years	912 (52.1%)	461 (56.4%)	451 (48.4%)	0.005	1 (ref.)		1 (ref.)	
	41-60 years	767 (43.9%)	329 (40.2%)	438 (47.0%)		1.36 (1.12, 1.65)	0.002	0.84 (0.58, 1.22)	0.363
	61-70 years	60 (3.4%)	26 (3.2%)	34 (3.7%)		1.34 (0.79, 2.26)	0.28	0.58 (0.26, 1.27)	0.173
	>70 years	10 (0.6%)	2 (0.2%)	8 (0.9%)		4.09 (0.86, 19.36)	0.076	1.25 (0.21, 7.25)	0.805
**Language, n (%)**	German	1467 (83.9%)	618 (75.6%)	849 (91.2%)	<0.001	1 (ref.)		1 (ref.)	
	French	213 (12.2%)	167 (20.4%)	46 (4.9%)		0.2 (0.14, 0.28)	<0.001	0.21 (0.15, 0.29)	<0.001
	Italian	69 (3.9%)	33 (4.0%)	36 (3.9%)		0.79 (0.49, 1.29)	0.35	0.77 (0.47, 1.26)	0.305
**Religion, n (%)**	Don't know / no information	79 (4.5%)	40 (4.9%)	39 (4.2%)	0.22	1 (ref.)		1 (ref.)	
	No religion	666 (38.1%)	300 (36.7%)	366 (39.3%)		1.24 (0.78, 1.98)	0.358	1.26 (0.78, 2.01)	0.344
	Reformed (Evangelical)	522 (29.8%)	247 (30.2%)	275 (29.5%)		1.19 (0.74, 1.92)	0.48	1.08 (0.67, 1.75)	0.743
	Catholic	464 (26.5%)	218 (26.7%)	246 (26.4%)		1.16 (0.72, 1.87)	0.548	1.1 (0.68, 1.79)	0.689
	Muslim	14 (0.8%)	11 (1.3%)	3 (0.3%)		0.28 (0.07, 1.08)	0.065	0.3 (0.08, 1.15)	0.078
	Other	4 (0.2%)	2 (0.2%)	2 (0.2%)		1.03 (0.14, 7.65)	0.98	1.04 (0.14, 7.81)	0.967
**Religiousness, n (%)**	Yes	493 (28.2%)	222 (27.1%)	271 (29.1%)	0.36	1.1 (0.89, 1.36)	0.388	1.01 (0.82, 1.25)	0.922
**What is your concept of the afterlife?, n (%)**	Don't know / no information	410 (23.4%)	173 (21.1%)	237 (25.5%)	0.019	1 (ref.)		1 (ref.)	
	Rebirth (reincarnation)	67 (3.8%)	35 (4.3%)	32 (3.4%)		0.69 (0.4, 1.16)	0.161	0.66 (0.39, 1.12)	0.121
	Only the body dies, the soul lives on	655 (37.4%)	334 (40.8%)	321 (34.5%)		0.7 (0.54, 0.9)	0.006	0.66 (0.51, 0.85)	0.002
	When a person dies, body and soul die. There is no survival.	617 (35.3%)	276 (33.7%)	341 (36.6%)		0.9 (0.7, 1.16)	0.422	0.9 (0.7, 1.16)	0.418
**Children, n (%)**	Yes	889 (50.8%)	433 (52.9%)	456 (49.0%)	0.099	0.84 (0.69, 1.01)	0.068	0.69 (0.55, 0.85)	0.001
**Nationality, n (%)**	Swiss	1455 (83.2%)	676 (82.6%)	779 (83.7%)	0.19	1 (ref.)		1 (ref.)	
	German	189 (10.8%)	86 (10.5%)	103 (11.1%)		1.12 (0.82, 1.53)	0.476	1.04 (0.77, 1.42)	0.785
	French	15 (0.9%)	12 (1.5%)	3 (0.3%)		0.22 (0.06, 0.77)	0.018	0.26 (0.07, 0.93)	0.039
	Italian	24 (1.4%)	11 (1.3%)	13 (1.4%)		1.02 (0.46, 2.3)	0.956	1.19 (0.53, 2.7)	0.675

	Austrian	22 (1.3%)	12 (1.5%)	10 (1.1%)		0.72 (0.31, 1.68)	0.449	0.71 (0.31, 1.67)	0.44
	Other	44 (2.5%)	21 (2.6%)	23 (2.5%)		0.95 (0.52, 1.73)	0.862	0.95 (0.52, 1.74)	0.862
**Swiss nationality, n (%)**	Yes	1465 (83.8%)	684 (83.6%)	781 (83.9%)	0.88	1.03 (0.8, 1.33)	0.81	0.99 (0.77, 1.28)	0.956
**Do you suffer or have you suffered from any of the following diseases?, n (%)**	Cancer (diagnosed within the past 3 years)	24 (1.4%)	7 (0.9%)	17 (1.8%)	0.082	2.15 (0.89, 5.22)	0.089	1.9 (0.78, 4.65)	0.157
	Cardiovascular disease (e.g. high blood pressure, heart attack, congestive heart failure)	126 (7.2%)	62 (7.6%)	64 (6.9%)	0.57	0.9 (0.63, 1.29)	0.569	0.77 (0.53, 1.12)	0.176
	Diabetes	16 (0.9%)	10 (1.2%)	6 (0.6%)	0.21	0.52 (0.19, 1.45)	0.213	0.46 (0.17, 1.28)	0.139
	Kidney disease (e.g. chronic kidney failure)	9 (0.5%)	5 (0.6%)	4 (0.4%)	0.60	0.7 (0.19, 2.62)	0.598	0.69 (0.18, 2.63)	0.59
	Lung or respiratory disease (e.g. asthma or smoker's lung)	108 (6.2%)	54 (6.6%)	54 (5.8%)	0.49	0.87 (0.59, 1.29)	0.488	0.9 (0.6, 1.33)	0.587
	Chronic infectious disease (e.g. HIV, hepatitis, tuberculosis)	5 (0.3%)	2 (0.2%)	3 (0.3%)	0.76	1.32 (0.22, 7.91)	0.762	1.27 (0.21, 7.71)	0.793
	Mental illness	49 (2.8%)	23 (2.8%)	26 (2.8%)	0.98	0.99 (0.56, 1.75)	0.981	1.05 (0.59, 1.87)	0.865
	Neurological diseases (e.g. multiple sclerosis, paralysis)	14 (0.8%)	7 (0.9%)	7 (0.8%)	0.81	0.88 (0.31, 2.51)	0.808	0.72 (0.25, 2.09)	0.544
	No previous illnesses	1197 (68.4%)	549 (67.1%)	648 (69.6%)	0.26	1.12 (0.92, 1.37)	0.264	1.2 (0.97, 1.47)	0.088
**Anxiety (GAD-2), n (%)**	Yes	105 (6.0%)	52 (6.4%)	53 (5.7%)	0.56	0.88 (0.59, 1.31)	0.526	0.9 (0.6, 1.34)	0.598
**Depression (PHQ-2), n (%)**	Yes	58 (3.3%)	28 (3.4%)	30 (3.2%)	0.82	0.93 (0.55, 1.57)	0.787	1 (0.59, 1.69)	0.996
**Health self-rating VAS [0-100], mean (SD)**		87.4 (11.6)	87.2 (11.9)	87.6 (11.2)	0.41	1 (0.99, 1.01)	0.46	1 (1, 1.01)	0.348
Experience with cardiac arrest									
**Have you ever been admitted to an intensive care unit?, n (%)**	Yes	117 (6.7%)	57 (7.0%)	60 (6.4%)	0.66	0.91 (0.62, 1.33)	0.623	0.88 (0.6, 1.29)	0.512
**Has someone very close to you ever been admitted to an intensive care unit?, n (%)**	Yes	1060 (60.6%)	497 (60.8%)	563 (60.5%)	0.90	0.99 (0.82, 1.21)	0.947	0.93 (0.76, 1.13)	0.468
**Have you ever witnessed a cardiopulmonary resuscitation?, n (%)**	Yes	1705 (97.5%)	793 (96.9%)	912 (98.0%)	0.18	1.55 (0.85, 2.84)	0.154	1.5 (0.82, 2.77)	0.191
**Have you ever been resuscitated?, n (%)**	Yes	18 (1.0%)	7 (0.9%)	11 (1.2%)	0.50	1.37 (0.53, 3.56)	0.513	1.55 (0.59, 4.04)	0.373
**Do you have an advance directive?, n (%)**	Yes	551 (32.4%)	201 (25.3%)	350 (38.6%)	<0.001	1.86 (1.51, 2.29)	<0.001	1.76 (1.43, 2.17)	<0.001
Estimated survival									
**Estimated IHCA survival [0-100%], mean (SD)**		27.6 (20.2)	29 (20.9)	26.3 (19.5)	0.005	0.99 (0.99, 1)	0.005	0.99 (0.99, 1)	0.001
**Estimated OHCA survival [0-100%], mean (SD)**		12.6 (11.9)	13.97 (13.1)	11.4 (10.6)	<0.001	0.98 (0.97, 0.99)	<0.001	0.98 (0.97, 0.99)	<0.001
**Estimated IHCA survival (categories), n (%)**	Correctly estimated (5% tolerance)	443 (25.3%)	197 (24.1%)	246 (26.4%)	0.044	1 (ref.)		1 (ref.)	

	Underestimated	474 (27.1%)	206 (25.2%)	268 (28.8%)		1.04 (0.8, 1.35)	0.758	1.07 (0.82, 1.39)	0.624
	Overestimated	832 (47.6%)	415 (50.7%)	417 (44.8%)		0.8 (0.64, 1.01)	0.065	0.79 (0.62, 0.99)	0.043
**Estimated OHCA survival (categories), n (%)**	Correctly estimated (5% tolerance)	1005 (57.5%)	451 (55.1%)	554 (59.5%)	0.001	1 (ref.)		1 (ref.)	
	Underestimated	218 (12.5%)	88 (10.8%)	130 (14.0%)		1.19 (0.88, 1.61)	0.25	1.17 (0.86, 1.58)	0.316
	Overestimated	526 (30.1%)	279 (34.1%)	247 (26.5%)		0.71 (0.57, 0.88)	0.002	0.68 (0.55, 0.84)	<0.001
Resuscitation preferences									
**Resuscitation preference regarding the case vignette, n (%)**	DNR yes	1484 (84.8%)	635 (77.6%)	849 (91.2%)	<0.001	2.98 (2.26, 3.95)	<0.001	2.99 (2.25, 3.96)	<0.001
**In case of a cardiac arrest: At what time-point without any treatment should resuscitation not be attempted anymore? (min), mean (SD)**		10.5 (7)	12.2 (6.9)	8.9 (6.7)	<0.001	0.94 (0.93, 0.96)	<0.001	0.93 (0.91, 0.94)	<0.001
**In case of a cardiac arrest: At what time-point without any treatment should resuscitation not be attempted anymore? (categories), n (%)**	0-5 min	496 (28.4%)	107 (13.1%)	389 (41.8%)	<0.001	1 (ref.)		1 (ref.)	
	>5-10 min	671 (38.4%)	345 (42.2%)	326 (35.0%)		0.48 (0.36, 0.63)	<0.001	0.27 (0.2, 0.35)	<0.001
	>10-15 min	295 (16.9%)	169 (20.7%)	126 (13.5%)		0.34 (0.24, 0.48)	<0.001	0.21 (0.15, 0.29)	<0.001
	>15-60 min	287 (16.4%)	197 (24.1%)	90 (9.7%)		0.31 (0.22, 0.46)	<0.001	0.13 (0.09, 0.18)	<0.001
**In the event of severe illness and respiratory failure, would you wish to be mechanically ventilated?, n (%)**	NO	1086 (62.1%)	477 (58.3%)	609 (65.4%)	0.002	1.33 (1.09, 1.63)	0.005	1.35 (1.11, 1.65)	0.002
**If you had to decide now: What option would you prefer?, n (%)**	Don't know / no information	20 (1.1%)	10 (1.2%)	10 (1.1%)	<0.001	1 (ref.)		1 (ref.)	
	Prolonging life is more important to me, even if it means more pain and discomfort	107 (6.1%)	77 (9.4%)	30 (3.2%)		0.39 (0.15, 1.03)	0.058	0.39 (0.14, 1.03)	0.057
	Alleviating pain and discomfort is more important to me, even if this might shorten life	1444 (82.6%)	633 (77.4%)	811 (87.1%)		1.29 (0.53, 3.12)	0.573	1.24 (0.51, 3.03)	0.633
	I'm not sure what I would choose	178 (10.2%)	98 (12.0%)	80 (8.6%)		0.82 (0.32, 2.06)	0.667	0.78 (0.31, 1.98)	0.601
**In case of imminent death: Where would you like to die?, n (%)**	Don't know / no information	210 (12.0%)	83 (10.1%)	127 (13.6%)	0.047	1 (ref.)		1 (ref.)	
	At home	915 (52.3%)	444 (54.3%)	471 (50.6%)		0.69 (0.51, 0.94)	0.019	0.69 (0.51, 0.94)	0.02
	At a relative's or friend's house	80 (4.6%)	37 (4.5%)	43 (4.6%)		0.86 (0.42, 1.74)	0.671	0.79 (0.47, 1.33)	0.372
	In a hospital	123 (7.0%)	65 (7.9%)	58 (6.2%)		0.58 (0.37, 0.91)	0.019	0.57 (0.36, 0.9)	0.016
	In a hospice	221 (12.6%)	90 (11.0%)	131 (14.1%)		0.95 (0.65, 1.4)	0.799	0.9 (0.61, 1.33)	0.602
	In a nursing home	n.a.	n.a.	n.a.	n.a.	n.a.	n.a.	n.a.	n.a.
	Doesn't matter	200 (11.4%)	99 (12.1%)	101 (10.8%)		0.67 (0.45, 0.99)	0.042	0.66 (0.44, 0.98)	0.04
Profession-related information									

**Profession, n (%)**	Physician	573 (34.2%)	210 (26.9%)	363 (40.6%)	<0.001	1 (ref.)		1 (ref.)	
	Nurse	314 (18.8%)	160 (20.5%)	154 (17.2%)		0.56 (0.42, 0.74)	<0.001	0.51 (0.39, 0.67)	<0.001
	Paramedic	787 (47.0%)	410 (52.6%)	377 (42.2%)		0.53 (0.43, 0.66)	<0.001	0.6 (0.48, 0.75)	<0.001
**Position, n (%)**	Resident	122 (23.4%)	35 (17.9%)	87 (26.6%)	0.057	1 (ref.)		1 (ref.)	
	Attending	178 (34.1%)	64 (32.8%)	114 (34.9%)		0.72 (0.44, 1.18)	0.189	0.5 (0.29, 0.86)	0.012
	Consultant	167 (32.0%)	71 (36.4%)	96 (29.4%)		0.54 (0.33, 0.9)	0.017	0.28 (0.14, 0.54)	<0.001
	Head of Department	55 (10.5%)	25 (12.8%)	30 (9.2%)		0.48 (0.25, 0.93)	0.031	0.2 (0.08, 0.48)	<0.001
**Type of Emergency Medical Service, n (%)**	Urban area	455 (57.2%)	238 (57.3%)	217 (57.1%)	0.74	1 (ref.)		1 (ref.)	
	Rural area	302 (38.0%)	155 (37.3%)	147 (38.7%)		1.03 (0.77, 1.38)	0.855	1.03 (0.76, 1.38)	0.864
	Air rescue	38 (4.8%)	22 (5.3%)	16 (4.2%)		0.83 (0.43, 1.6)	0.573	0.79 (0.4, 1.55)	0.489
**Leadership position, n(%)**	Yes	651 (37.2%)	314 (38.4%)	337 (36.2%)	0.34	0.93 (0.73, 1.17)	0.521	0.83 (0.67, 1.02)	0.082
**Profession experience (years), mean (SD)**		14.3 (10.4)	13.5 (10.3)	15 (10.5)	0.003	1.01 (1, 1.02)	0.004	0.98 (0.96, 1)	0.027
**Number of CPRs**	None	19 (1.1%)	12 (1.5%)	7 (0.8%)	0.29	1 (ref.)		1 (ref.)	
	1 to 5	292 (16.7%)	146 (17.8%)	146 (15.7%)		1.71 (0.66, 4.48)	0.271	1.58 (0.6, 4.16)	0.353
	6 to 10	n.a.	n.a.	n.a.	n.a.	n.a.	n.a.	n.a.	n.a.
	11 to 20	249 (14.2%)	116 (14.2%)	133 (14.3%)		2.1 (0.8, 5.52)	0.134	1.79 (0.68, 4.74)	0.242
	21 to 50	1189 (68.0%)	544 (66.5%)	645 (69.3%)		2.04 (0.8, 5.21)	0.138	1.67 (0.64, 4.36)	0.292
	>50	n.a.	n.a.	n.a.	n.a.	n.a.	n.a.	n.a.	n.a.

*adjusted for age and self-reported gender
**With independence in activities of daily living (CPC 1-2).
Abbreviations: CPC , cerebral performance category scale; CPR, cardiopulmonary resuscitation; DNR, do-not-resuscitate; IHCA, in-hospital cardiac arrest; OHCA, out-of-hospital cardiac arrest; OR, odds ratio; ref., reference value; SD, standard deviation VAS, visual analogue scale.

Supplement 5. Interprofessional differences

		All	Physicians	Nurses	Paramedics	*p-value*
**n**		1722	580	330	812	
Baseline Characteristics						
**Gender, n (%)**	Male	936 (54.4%)	343 (59.1%)	83 (25.2%)	510 (62.8%)	<0.001
**Age, mean (SD)**		41.4 (10.5)	44.3 (10.5)	42.9 (10.9)	38.7 (9.6)	<0.001
**Age categories, n (%)**	≤40 years	903 (52.4%)	239 (41.2%)	154 (46.7%)	510 (62.8%)	<0.001
	41-60 years	754 (43.8%)	300 (51.7%)	164 (49.7%)	290 (35.7%)	
	61-70 years	58 (3.4%)	35 (6.0%)	11 (3.3%)	12 (1.5%)	
	>70 years	7 (0.4%)	6 (1.0%)	1 (0.3%)	0 (0.0%)	
**Language, n (%)**	German	1443 (83.8%)	503 (86.7%)	307 (93.0%)	633 (78.0%)	<0.001
	French	212 (12.3%)	55 (9.5%)	19 (5.8%)	138 (17.0%)	
	Italian	67 (3.9%)	22 (3.8%)	4 (1.2%)	41 (5.0%)	
**Religion, n (%)**	Don't know / no information	77 (4.5%)	25 (4.3%)	10 (3.0%)	42 (5.2%)	0.041
	No religion	678 (39.4%)	229 (39.5%)	120 (36.4%)	329 (40.5%)	
	Reformed (Evangelical)	481 (27.9%)	154 (26.6%)	101 (30.6%)	226 (27.8%)	
	Catholic	468 (27.2%)	165 (28.4%)	92 (27.9%)	211 (26.0%)	
	Muslim	15 (0.9%)	4 (0.7%)	7 (2.1%)	4 (0.5%)	
	Other	3 (0.2%)	3 (0.5%)	0 (0.0%)	0 (0.0%)	
**Religiousness, n (%)**	Yes	495 (28.7%)	179 (30.9%)	114 (34.5%)	202 (24.9%)	0.002
**What is your concept of the afterlife?, n (%)**	Don't know / no information	426 (24.7%)	131 (22.6%)	85 (25.8%)	210 (25.9%)	<0.001
	Rebirth (reincarnation)	67 (3.9%)	9 (1.6%)	10 (3.0%)	48 (5.9%)	
	Only the body dies, the soul lives on	613 (35.6%)	164 (28.3%)	143 (43.3%)	306 (37.7%)	
	When a person dies, body and soul die. There is no survival.	616 (35.8%)	276 (47.6%)	92 (27.9%)	248 (30.5%)	
**Children, n (%)**	Yes	871 (50.6%)	326 (56.2%)	151 (45.8%)	394 (48.5%)	0.003
**Nationality, n (%)**	Swiss	1426 (82.8%)	448 (77.2%)	235 (71.2%)	743 (91.5%)	<0.001
	German	190 (11.0%)	91 (15.7%)	65 (19.7%)	34 (4.2%)	
	French	14 (0.8%)	2 (0.3%)	5 (1.5%)	7 (0.9%)	
	Italian	26 (1.5%)	12 (2.1%)	4 (1.2%)	10 (1.2%)	
	Austrian	24 (1.4%)	7 (1.2%)	14 (4.2%)	3 (0.4%)	

	Other	42 (2.4%)	20 (3.4%)	7 (2.1%)	15 (1.8%)	
**Swiss nationality, n (%)**	Yes	1434 (83.3%)	451 (77.8%)	236 (71.5%)	747 (92.0%)	<0.001
**Do you suffer or have you suffered from any of the following diseases?, n (%)**	Cancer (diagnosed within the past 3 years)	23 (1.3%)	11 (1.9%)	4 (1.2%)	8 (1.0%)	0.34
	Cardiovascular disease (e.g. high blood pressure, heart attack, congestive heart failure)	125 (7.3%)	53 (9.1%)	23 (7.0%)	49 (6.0%)	0.087
	Diabetes	16 (0.9%)	8 (1.4%)	1 (0.3%)	7 (0.9%)	0.26
	Kidney disease (e.g. chronic kidney failure)	8 (0.5%)	2 (0.3%)	1 (0.3%)	5 (0.6%)	0.68
	Lung or respiratory disease (e.g. asthma or smoker's lung)	108 (6.3%)	34 (5.9%)	27 (8.2%)	47 (5.8%)	0.28
	Chronic infectious disease (e.g. HIV, hepatitis, tuberculosis)	5 (0.3%)	1 (0.2%)	1 (0.3%)	3 (0.4%)	0.8
	Mental illness	48 (2.8%)	15 (2.6%)	9 (2.7%)	24 (3.0%)	0.92
	Neurological diseases (e.g. multiple sclerosis, paralysis)	14 (0.8%)	4 (0.7%)	6 (1.8%)	4 (0.5%)	0.071
	No previous illnesses	1218 (70.7%)	398 (68.6%)	219 (66.4%)	601 (74.0%)	0.014
**Anxiety (GAD-2), n (%)**	Yes	108 (6.3%)	38 (6.6%)	22 (6.7%)	48 (5.9%)	0.84
**Depression (PHQ-2), n (%)**	Yes	58 (3.4%)	20 (3.4%)	14 (4.2%)	24 (3.0%)	0.55
**Health self-rating VAS [0-100], mean (SD)**		87.4 (11.7)	87.1 (12.7)	86.86 (12)	87.9 (10.7)	0.28
Experience with cardiac arrest						
**Have you ever been admitted to an intensive care unit?, n (%)**	Yes	119 (6.9%)	36 (6.2%)	22 (6.7%)	61 (7.5%)	0.63
**Has someone very close to you ever been admitted to an intensive care unit?, n (%)**	Yes	1033 (60.0%)	333 (57.4%)	193 (58.5%)	507 (62.4%)	0.14
**Have you ever witnessed a cardiopulmonary resuscitation?, n (%)**	Yes	1678 (97.4%)	571 (98.4%)	319 (96.7%)	788 (97.0%)	0.16
**Have you ever been resuscitated?, n (%)**	Yes	17 (1.0%)	3 (0.5%)	3 (0.9%)	11 (1.4%)	0.29
**Do you have an advance directive?, n (%)**	Yes	548 (31.9%)	163 (28.2%)	126 (38.3%)	259 (32.0%)	0.007
Estimated survival*						
**Estimated IHCA survival [0-100%], mean (SD)**		27.6 (20.5)	25.7 (17.3)	39.4 (24.5)	24.2 (19.1)	<0.001
**Estimated OHCA survival [0-100%], mean (SD)**		12.6 (12.1)	10.7 (9.2)	19.3 (16.3)	11.3 (11.1)	<0.001
**Estimated IHCA survival (categories), n (%)**	Correctly estimated (5% tolerance)	448 (26.0%)	184 (31.7%)	48 (14.5%)	216 (26.6%)	<0.001
	Underestimated	481 (27.9%)	137 (23.6%)	57 (17.3%)	287 (35.3%)	
	Overestimated	793 (46.1%)	259 (44.7%)	225 (68.2%)	309 (38.1%)	

**Estimated OHCA survival (categories), n (%)**	Correctly estimated (5% tolerance)	985 (57.2%)	358 (61.7%)	126 (38.2%)	501 (61.7%)	<0.001
	Underestimated	221 (12.8%)	81 (14.0%)	28 (8.5%)	112 (13.8%)	
	Overestimated	516 (30.0%)	141 (24.3%)	176 (53.3%)	199 (24.5%)	
Resuscitation preferences						
**Resuscitation preference regarding the case vignette, n (%)**	DNR yes	1464 (85.0%)	476 (82.1%)	293 (88.8%)	695 (85.6%)	0.02
**Personal resuscitation preference, n (%)**	DNR yes	941 (54.6%)	370 (63.8%)	170 (51.5%)	401 (49.4%)	<0.001
**In case of a cardiac arrest: At what time-point without any treatment should resuscitation not be attempted anymore? (min), mean (SD)**		10.4 (7)	10.9 (7.5)	10.4 (7.4)	10.1 (6.5)	0.15
**In case of a cardiac arrest: At what time-point without any treatment should resuscitation not be attempted anymore? (categories), n (%)**	0-5 min	504 (29.3%)	164 (28.3%)	104 (31.5%)	236 (29.1%)	0.44
	>5-10 min	644 (37.4%)	219 (37.8%)	111 (33.6%)	314 (38.7%)	
	>10-15 min	295 (17.1%)	92 (15.9%)	64 (19.4%)	139 (17.1%)	
	>15-60 min	279 (16.2%)	105 (18.1%)	51 (15.5%)	123 (15.1%)	
**In the event of severe illness and respiratory failure, would you wish to be mechanically ventilated?, n (%)**	NO	1066 (61.9%)	271 (46.7%)	213 (64.5%)	582 (71.7%)	<0.001
**If you had to decide now: What option would you prefer?, n (%)**	Don't know / no information	21 (1.2%)	11 (1.9%)	2 (0.6%)	8 (1.0%)	<0.001
	Prolonging life is more important to me, even if it means more pain and discomfort	108 (6.3%)	62 (10.7%)	14 (4.2%)	32 (3.9%)	
	Alleviating pain and discomfort is more important to me, even if this might shorten life	1414 (82.1%)	431 (74.3%)	288 (87.3%)	695 (85.6%)	
	I'm not sure what I would choose	179 (10.4%)	76 (13.1%)	26 (7.9%)	77 (9.5%)	
**In case of imminent death: Where would you like to die?, n (%)**	Don't know / no information	214 (12.4%)	74 (12.8%)	34 (10.3%)	106 (13.1%)	<0.001
	At home	929 (53.9%)	304 (52.4%)	159 (48.2%)	466 (57.4%)	
	At a relative's or friend's house	37 (2.1%)	12 (2.1%)	5 (1.5%)	20 (2.5%)	
	In a hospital	124 (7.2%)	50 (8.6%)	23 (7.0%)	51 (6.3%)	
	In a hospice	218 (12.7%)	61 (10.5%)	70 (21.2%)	87 (10.7%)	
	In a nursing home					
	Doesn't matter	200 (11.6%)	79 (13.6%)	39 (11.8%)	82 (10.1%)	
Profession-related information						
**Position, n (%)**	Resident	125 (21.6%)	125 (21.6%)	n.a.	n.a.	
	Attending	194 (33.4%)	194 (33.4%)	n.a.	n.a.	
	Consultant	191 (32.9%)	191 (32.9%)	n.a.	n.a.	

	Head of Department	70 (12.1%)	70 (12.1%)	n.a.	n.a.	
**Type of Emergency Medical Service, n (%)**	Urban area	462 (56.9%)	n.a.	n.a.	462 (56.9%)	
	Rural area	310 (38.2%)	n.a.	n.a.	310 (38.2%)	
	Air rescue	40 (4.9%)	n.a.	n.a.	40 (4.9%)	
**Leadership position, n(%)**	Yes	647 (37.6%)	206 (35.5%)	137 (41.5%)	304 (37.4%)	0.2
**Professional experience (years), mean (SD)**		14.2 (10.4)	17 (10.2)	17 (11.1)	11.1 (9.3)	<0.001
**Number of CPRs**	None	20 (1.2%)	8 (1.4%)	9 (2.7%)	3 (0.4%)	<0.001
	1 to 5	296 (17.2%)	60 (10.3%)	125 (37.9%)	111 (13.7%)	
	6 to 10					
	11 to 20	240 (13.9%)	47 (8.1%)	55 (16.7%)	138 (17.0%)	
	21 to 50	1166 (67.7%)	465 (80.2%)	141 (42.7%)	560 (69.0%)	

*With independence in activities of daily living (CPC 1-2).
Abbreviations: CPC , cerebral performance category scale; CPR, cardiopulmonary resuscitation; DNR, do-not-resuscitate; IHCA, in-hospital cardiac arrest; OHCA, out-of-hospital cardiac arrest; OR, odds ratio; ref., reference value; SD, standard deviation VAS, visual analogue scale.

Supplement 6. Differences between the Swiss general population and healthcare professionals

		All	General Population	Healthcare Professionals	*p-value*
**n**		2847	1044	1803	
Baseline Characteristics					
**Gender, n (%)**	Male	1503 (52.9%)	528 (50.6%)	975 (54.3%)	0.056
**Age, mean (SD)**		42.9 (13.1)	45.4 (16.3)	41.44 (10.6)	<0.001
**Age categories, n (%)**	≤40 years	1375 (48.3%)	432 (41.4%)	943 (52.3%)	<0.001
	41-60 years	1202 (42.2%)	414 (39.7%)	788 (43.7%)	
	61-70 years	183 (6.4%)	121 (11.6%)	62 (3.4%)	
	>70 years	87 (3.1%)	77 (7.4%)	10 (0.6%)	
**Language, n (%)**	German	2238 (78.6%)	719 (68.9%)	1519 (84.2%)	<0.001
	French	462 (16.2%)	247 (23.7%)	215 (11.9%)	
	Italian	147 (5.2%)	78 (7.5%)	69 (3.8%)	
**Religion, n (%)**	Don't know / no information	97 (3.5%)	18 (1.7%)	79 (4.6%)	<0.001
	No religion	975 (35.4%)	290 (27.8%)	685 (40.1%)	
	Reformed (Evangelical)	753 (27.4%)	305 (29.2%)	448 (26.2%)	
	Catholic	849 (30.8%)	371 (35.5%)	478 (28.0%)	
	Muslim	29 (1.1%)	14 (1.3%)	15 (0.9%)	
	Other	50 (1.8%)	46 (4.4%)	4 (0.2%)	
**Religiousness, n (%)**	Yes	933 (35.0%)	437 (42.9%)	496 (30.1%)	<0.001
**What is your concept of the afterlife?, n (%)**	Don't know / no information	565 (20.5%)	131 (12.5%)	434 (25.4%)	<0.001
	Rebirth (reincarnation)	147 (5.3%)	80 (7.7%)	67 (3.9%)	
	Only the body dies, the soul lives on	1035 (37.6%)	454 (43.5%)	581 (34.0%)	
	When a person dies, body and soul die. There is no survival.	1006 (36.5%)	379 (36.3%)	627 (36.7%)	
**Do you suffer or have you suffered from any of the following diseases?, n (%)**	Cancer (diagnosed within the past 3 years)	58 (2.0%)	34 (3.3%)	24 (1.3%)	<0.001
	Cardiovascular disease (e.g. high blood pressure, heart attack, congestive heart failure)	280 (9.8%)	152 (14.6%)	128 (7.1%)	<0.001
	Diabetes	56 (2.0%)	40 (3.8%)	16 (0.9%)	<0.001
	Kidney disease (e.g. chronic kidney failure)	30 (1.1%)	21 (2.0%)	9 (0.5%)	<0.001
	Lung or respiratory disease (e.g. asthma or smoker's lung)	177 (6.2%)	66 (6.3%)	111 (6.2%)	0.86
	Chronic infectious disease (e.g. HIV, hepatitis, tuberculosis)	11 (0.4%)	6 (0.6%)	5 (0.3%)	0.22
	Mental illness	123 (4.3%)	74 (7.1%)	49 (2.7%)	<0.001

	Neurological diseases (e.g. multiple sclerosis, paralysis)	35 (1.2%)	21 (2.0%)	14 (0.8%)	0.004
	No previous illnesses	1867 (65.6%)	629 (60.2%)	1238 (68.7%)	<0.001
	Other	67 (2.4%)	67 (6.4%)	0 (0.0%)	<0.001
	Don't know / no information	50 (1.8%)	50 (4.8%)	0 (0.0%)	<0.001
**Anxiety (GAD-2), n (%)**	Yes	229 (8.3%)	121 (11.6%)	108 (6.3%)	<0.001
**Depression (PHQ-2), n (%)**	Yes	151 (5.5%)	93 (8.9%)	58 (3.4%)	<0.001
**Health self-rating VAS [0-100], mean (SD)**		84.2 (14.1)	78.9 (16)	87.5 (11.8)	<0.001
Experience with cardiac arrest					
**Have you ever been admitted to an intensive care unit?, n (%)**	Yes	236 (8.6%)	114 (11.0%)	122 (7.1%)	<0.001
**Has someone very close to you ever been admitted to an intensive care unit?, n (%)**	Yes	1594 (58.3%)	583 (56.5%)	1011 (59.4%)	0.13
**Have you ever witnessed a cardiopulmonary resuscitation?, n (%)**	Yes	1822 (66.7%)	172 (16.6%)	1650 (97.3%)	<0.001
**Have you ever been resuscitated?, n (%)**	Yes	42 (1.5%)	24 (2.3%)	18 (1.1%)	0.009
**Do you have an advance directive?, n (%)**	Yes	846 (30.6%)	283 (28.0%)	563 (32.1%)	0.025
Estimated survival*					
**Estimated IHCA survival [0-100%], mean (SD)**		32.8 (23.4)	41.6 (25.4)	27.7 (20.6)	<0.001
**Estimated OHCA survival [0-100%], mean (SD)**		31 (30.2)	62.9 (25.1)	12.7 (12.1)	<0.001
**Estimated IHCA survival (categories), n (%)**	Correctly estimated (5% tolerance)	594 (21.4%)	139 (13.6%)	455 (25.9%)	<0.001
	Underestimated	668 (24.0%)	177 (17.3%)	491 (27.9%)	
	Overestimated	1517 (54.6%)	706 (69.1%)	811 (46.2%)	
**Estimated OHCA survival (categories), n (%)**	Correctly estimated (5% tolerance)	1055 (38.1%)	51 (5.0%)	1004 (57.1%)	<0.001
	Underestimated	231 (8.3%)	6 (0.6%)	225 (12.8%)	
	Overestimated	1485 (53.6%)	957 (94.4%)	528 (30.1%)	
Resuscitation preferences					
**Resuscitation preference regarding the case vignette, n (%)**	DNR yes	1955 (68.7%)	423 (40.5%)	1532 (85.0%)	<0.001
**Personal resuscitation preference, n (%)**	DNR yes	1140 (41.0%)	209 (20.3%)	931 (53.2%)	<0.001
**In case of a cardiac arrest: At what time-point without any treatment should resuscitation not be attempted anymore? (min), mean (SD)**		12.6 (10.6)	18.3 (13.8)	10 (7.4)	<0.001

**In the event of severe illness and respiratory failure, would you wish to be mechanically ventilated?, n (%)**	NO	1683 (61.9%)	652 (63.4%)	1031 (61.0%)	0.22
**If you had to decide now: What option would you prefer?, n (%)**	Don't know / no information	29 (1.0%)	7 (0.7%)	22 (1.3%)	<0.001
	Prolonging life is more important to me, even if it means more pain and discomfort	171 (6.1%)	62 (5.9%)	109 (6.2%)	
	Alleviating pain and discomfort is more important to me, even if this might shorten life	2182 (77.9%)	737 (70.6%)	1445 (82.2%)	
	I'm not sure what I would choose	419 (15.0%)	238 (22.8%)	181 (10.3%)	
**In case of imminent death: Where would you like to die?, n (%)**	Don't know / no information	253 (9.2%)	30 (3.0%)	223 (12.7%)	<0.001
	At home	1543 (55.9%)	596 (59.5%)	947 (53.9%)	
	At a relative's or friend's house	51 (1.8%)	14 (1.4%)	37 (2.1%)	
	In a hospital	214 (7.8%)	88 (8.8%)	126 (7.2%)	
	In a hospice	284 (10.3%)	61 (6.1%)	223 (12.7%)	
	In a nursing home	10 (0.4%)	10 (1.0%)	0 (0.0%)	
	Doesn't matter	403 (14.6%)	202 (20.2%)	201 (11.4%)	

*With independence in activities of daily living (CPC 1-2).
Abbreviations: CPC , cerebral performance category scale; CPR, cardiopulmonary resuscitation; DNR, do-not-resuscitate; IHCA, in-hospital cardiac arrest; OHCA, out-of-hospital cardiac arrest; OR, odds ratio; ref., reference value; SD, standard deviation VAS, visual analogue scale.
